# Differential gene regulatory pattern in the human brain from schizophrenia using transcriptomic-causal network

**DOI:** 10.1186/s12859-020-03753-6

**Published:** 2020-10-21

**Authors:** Akram Yazdani, Raul Mendez-Giraldez, Azam Yazdani, Michael R. Kosorok, Panos Roussos

**Affiliations:** 1 Department of Pharmacotherapy and Experimental Therapeutics, Eshelman School of Pharmacy, University of North Carolina at Chapel Hill, 120 Mason Farm Road, Genetic Medicine Building, CB#7361, Chapel Hill, NC 27599-7264 USA; 2 Lineberger Comprehensive Cancer Center, School of Medicine, University of North Carolina at Chapel Hill, Chapel Hill, NC 27514 USA; 3 Department of Anesthesiology, Perioperative and Pain Medicine, Brigham and Women’s Hospital, Harvard Medical School, Boston, MA 02115 USA; 4 Department of Biostatistics, University of North Carolina at Chapel Hill, Chapel Hill, NC 27599 USA; 5 Department of Psychiatry, Pamela Sklar Division of Psychiatric Genomics and Friedman Brain Institute, Icahn School of Medicine At Mount Sinai, Hess CSM Building Floor 9 Room 107, 1470 Madison Ave, New York, NY 10029 USA; 6 Mental Illness Research Education and Clinical Center (MIRECC), James J. Peters VA Medical Center, Bronx, NY 10468 USA

**Keywords:** Bayesian causal network, Mendelian randomization, Data integration, Transcriptomic, *Cis/trans*-regulatory factors, Schizophrenia

## Abstract

**Background:**

Common and complex traits are the consequence of the interaction and regulation of multiple genes simultaneously, therefore characterizing the interconnectivity of genes is essential to unravel the underlying biological networks. However, the focus of many studies is on the differential expression of individual genes or on co-expression analysis.

**Methods:**

Going beyond analysis of one gene at a time, we systematically integrated transcriptomics, genotypes and Hi-C data to identify interconnectivities among individual genes as a causal network. We utilized different machine learning techniques to extract information from the network and identify differential regulatory pattern between cases and controls. We used data from the Allen Brain Atlas for replication.

**Results:**

Employing the integrative systems approach on the data from CommonMind Consortium showed that gene transcription is controlled by genetic variants proximal to the gene (cis-regulatory factors), and transcribed distal genes (trans-regulatory factors). We identified differential gene regulatory patterns in SCZ-cases versus controls and novel SCZ-associated genes that may play roles in the disorder since some of them are primary expressed in human brain. In addition, we observed genes known associated with SCZ are not likely (OR = 0.59) to have high impacts (degree > 3) on the network.

**Conclusions:**

Causal networks could reveal underlying patterns and the role of genes individually and as a group. Establishing principles that govern relationships between genes provides a mechanistic understanding of the dysregulated gene transcription patterns in SCZ and creates more efficient experimental designs for further studies. This information cannot be obtained by studying a single gene at the time.

## Background

Differences in gene expression are likely to underpin much of human diversity, including psychiatric disorders such as schizophrenia (SCZ). SCZ is a severe, complex, and heritable psychiatric disorder with a worldwide prevalence of 1%, characterized by abnormalities in thought and cognition. Recent studies on SCZ, which comprise linkage scans and their meta-analyses, candidate gene association analysis, differential expression analysis and genome-wide association studies, investigated genes/markers and chromosomal regions for SCZ [1–5]. These studies showed that SCZ disorder involves changes in multiple genes [6, 7]. Fortunately, recent technologies of high-throughput sequencing provide opportunities to develop new tools for understanding the functional status of genes from a systemic perspective [8, 9] and uncovering the underlying SCZ processes.

Co-expression network analysis is one of the approaches that aim to infer gene function from genome-wide gene expression data. However, this kind of networks reveals groups of co-activated genes based on pairwise correlations, they do not normally confer information about causality or distinguish between regulatory and regulated genes [10]. In addition, they face limited success at gene functional inference due to finding many dependencies [11]. To reduce the dependencies, Bayesian networks can be constructed which are based on conditional probabilities and represent in/dependence properties between two genes after conditioning on the other genes in the study. Therefore, Bayesian networks are more informative and sparser compared to co-expression networks, especially in large-scale omics data. Although Bayesian networks can be directed, the directions may not be robustly identified. In order to find robust directions, causal networks are suggested. A causal network is a Bayesian network augmented with Mendelian randomization principles that provide stable directionality [9] with causal interpretation.

Causal networks are established in the recognition of the hierarchical structure of the biological systems and provide a better understanding of the regulatory patterns. In addition, causal networks result in finding novel intervention targets and revealing underlying pathways with reproducible results [12]. Studying transcriptomic-causal networks may lead to identification of differential gene regulation patterns, unveil the mechanisms that control the transcription of the SCZ-associated genes, and narrow down the search space for finding *trans*-regulatory elements. Considering all together, transcriptomic-causal networks provide a mechanistic understanding of disease processes and facilitate more efficient experimental design for further studies [8], which cannot be obtained using common approaches in transcriptomic data analysis such as differential expression (DE) and transcriptome-wide association study (TWAS). We compare utilities of transcriptomic-causal network with DE and TWAS in the discussion.

To divulge underlying relationships of SCZ related genes, we use genotypes and Hi-C data and build a causal network over RNA-seq data from the dorsolateral prefrontal cortex of the CommonMind Consortium (CMC) in the study of SCZ and use the data from the Allen Brain Atlas [13] for replication. We utilize some properties of the transcriptomic-causal networks to provide a mechanistic understanding of gene transcription and SCZ processes in the brain. Our analysis reveals that highly connected genes in the network (degree ≥ 3) are less likely (OR = 0.5) to be affected by genetic variants. These genes are more often found regulated by or regulating other distal genes as *trans-*regulatory factors. Furthermore, utilizing machine learning approaches to extract information from the network, we identify genes that are differentially regulated in SCZ-cases versus controls and essential genes in human brain, which shows similar impact on the network in both cases and controls. In addition, by mapping the genes previously reported as SCZ-associated genes on the network, we observe less than 10% of the SCZ-associated genes have high impacts on the networks. This result can be an explanation for the small effect of most SCZ-associated genes on the SCZ pathophysiology.

To the best of our knowledge, this approach of integrating DNA and gene expression and Hi-C data to analyze transcriptomic data systematically is a novel approach. In addition, extracting information from causal networks through statistical techniques and machine learning algorithms to compare case and control data is for the first time in the context of gene expression and systems approaches.

### Data

#### RNA-seq

Gene expression profiles (RNA-seq) from the dorsolateral prefrontal cortex of 258 cases with SCZ and 279 controls are available on CMC website (https://www.synapse.org/CMC), and the details on QC and normalization can be found in [14]. Following data normalization, 16,423 genes (based on Ensemble models) were expressed at levels sufficient (at least 1 CPM, count per million, in at least 50% of the individuals) for analysis, of which 14,222 were protein-coding. Covariates for RNA integrity (RIN), library batch, institution, diagnosis, age at death, genetic ancestry, post-mortem interval and sex together explained a substantial fraction (42%) of the average variance of gene transcription and were thus employed to adjust the data for the analyses [14]. Moreover, we use RNA-seq data available in the Allen Brain Atlas [13] for replication. We select 25 white matter in control samples since degeneration in this tissue is related to reduce prefrontal cortex activation [15].

#### Genotype data

Samples were genotyped on the Illumina Infinium HumanOmniExpressExome array (958,178 single-nucleotide polymorphisms, or SNPs). These genotypes were used to estimate the ancestry of the samples and to ensure sample identity across DNA and RNA experiments [14]. The original distribution of ethnicities in CMC includes 80.7% Caucasian and 19.3% from other ethnicities. To prevent the study from being confounded due to different ethnicities, we include only Caucasians (209 and 206 samples in SCZ and control group respectively).

#### Hi-C data

Hi- C data can be generated using Chromosome conformation capture techniques, which are a set of molecular biology methods used to analyze the spatial organization of chromatin in cells. These methods quantify the number of interactions between genomic loci that are nearby in 3-D space but may be separated by many nucleotides in the linear genome [16]. Hi-C data from [17] is used in this study. The Hi-C libraries were constructed from mid-gestation developing human cerebral cortex during the peak of neurogenesis and migration from two major zones: the cortical and subcortical plate, consisting primarily of post-mitotic neurons and the germinal zone, containing primarily mitotically active neural progenitors.

### Overview of the method

**Fig. 1:**
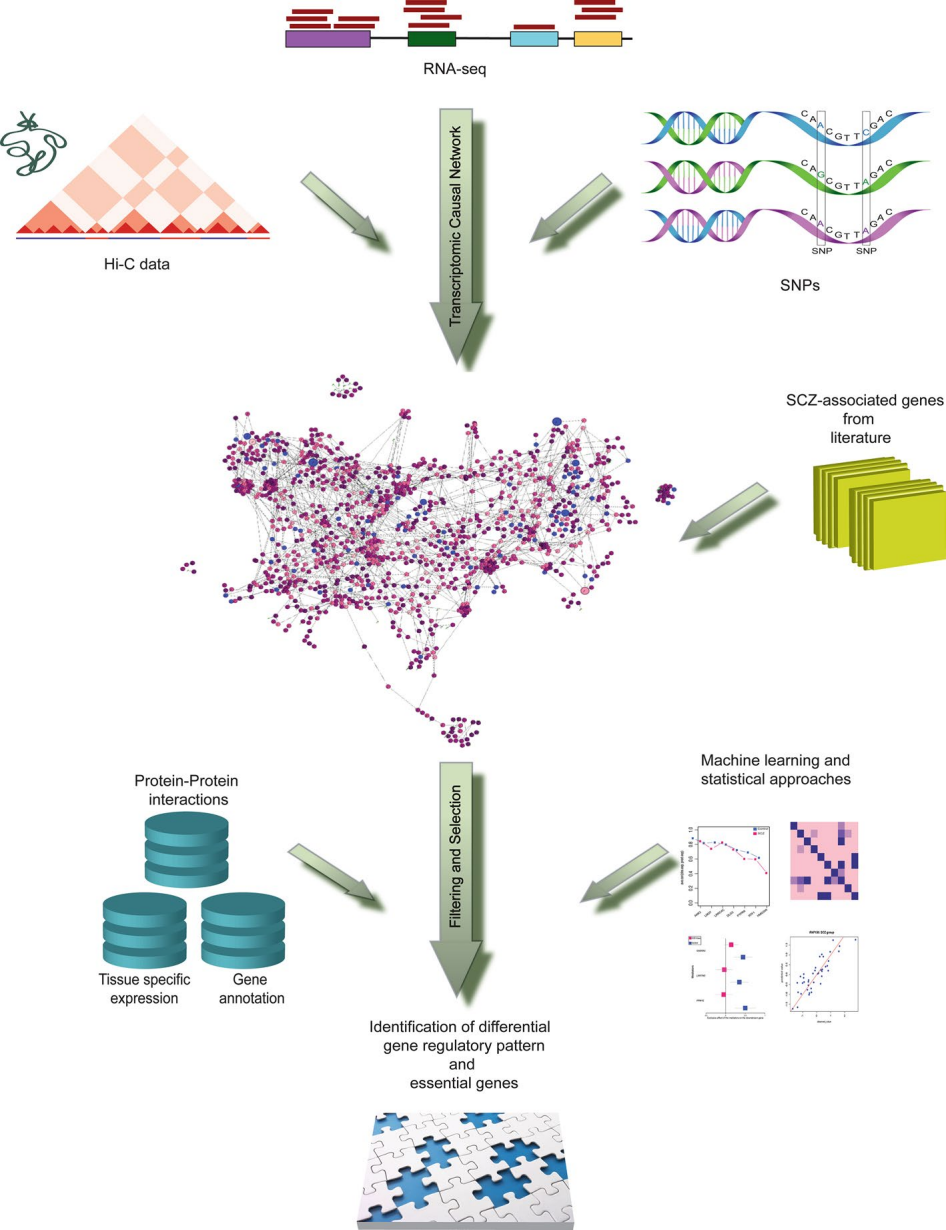
Schematic representation of our approach

Figure 1 provides a schematic representation of the approach. Each step is briefly explained in the following and detailed in the method section.

#### Step-1: Building transcriptomic-causal network

We integrate information from genotype, transcriptomics data, and Hi-C to construct transcriptomic-causal network, according to which the genetic variations affect gene expressions unidirectionally. We assess the quality of the fit by employing Hamming distance [18] and test the stability of the network using a permutation test.

#### Step-2: Mapping SCZ-associated genes on the network

To extract information from the network with focus of SCZ, we map the SCZ-associated genes, previously reported in the literature, on the network.

#### Step-3: Utilizing machine learning and statistical approaches to extract network properties

The causal network properties measure the impact of each gene individually (such as out-degree and in-degree) and genes as a group (such as modules) as well as pathways that represent how the effect of an intervention spreads across the system/transcription network.

**Fig. 2:**
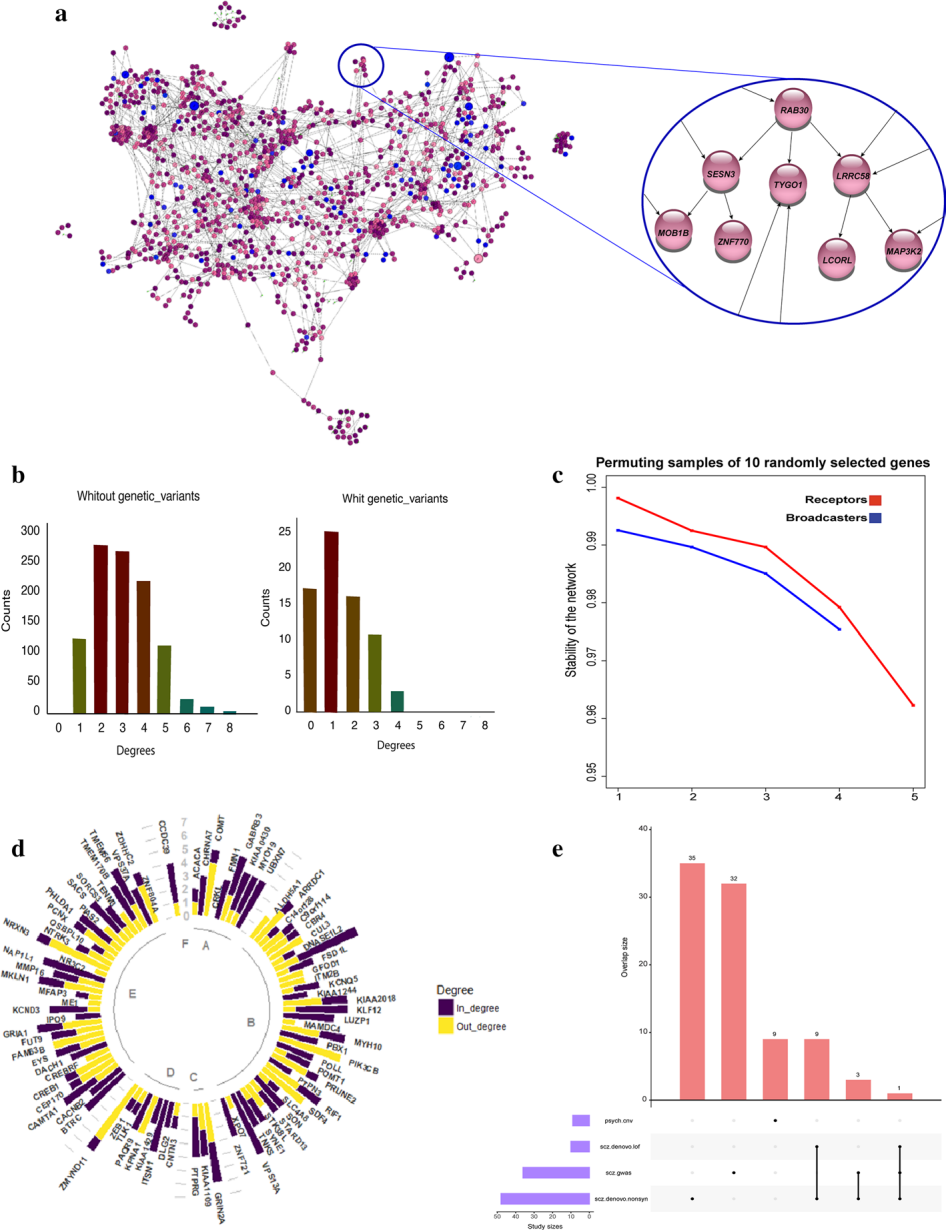
**a** Identified transcriptomic-causal network and a close-up. Each node represents a gene. Genes associated with SCZ are depicted in blue. **b** Distribution of the connectivity of the genes with genetic variants (left) and the genes without genetic variants (right) in the network. **c** Stability of the network after permutation of broadcaster genes (blue curve) and receptors genes (red curve). **d** Presentation of in-degree and out-degrees of genes associated with SCZ in the network. Single color bars in yellow or purple are corresponding to broadcaster and receptor genes respectively while bars with both colors are representing mediator genes. **e** Venn diagram groups SCZ-associated genes based on the type of studies that previously found their association (psych.cnv: Psychiatric study of copy number variation; SCZ.denovo.nonsyn: SCZ study of non-synonymous de novo mutations; SCZ.gwas: SCZ study of genome wide association studies; SCZ.denova.lof: SCZ study of Loss-of-function de novo mutations)

The *out-degree* of a gene refers to the number of downstream genes directly affected by the gene of interest, whereas *in-degree* of a gene refers to the number of upstream genes affected the gene of interest. Based on their connectivity, genes can be classified as broadcasters if they have non-zero out-degree and zero in-degree; receptors if they have non-zero in-degree and zero out-degree; and mediator if they have both non-zero out- and in-degrees. For instance, *RAB30* in Fig. 2a (closeup) is a broadcaster gene; *ZNF770* is a receptor gene; *TYGO1* is a mediator.

The effect of a gene can be propagated downstream until it reaches a receptor on a given path called *pathway*. Genes with a high number of out-degree and long pathways have a high impact on the network and hence are considered as “*cores*”. We define a module as a subnetwork that includes a core and genes highly influenced by the core directly or indirectly. The boundaries of the modules are made of receptor genes that prevent the effect of the core distributs beyond these genes.

Utilizing these properties, we investigate the relationship between connectivity of the genes in the network and other properties such as being SCZ associated, differentially expressed, and loss of function intolerant. We also assess the impact of the cores on other genes in the corresponding modules using a linear regression model and cross-validation.

#### Step-4: Identifying differential regulatory patterns between SCZ-cases and controls, essential genes for the brain function and novel genes putatively related to SCZ

To estimate differential regulatory patterns between SCZ-cases and controls, we seek genes with a high degree of connectivity (degree ≥ 3) in the networks whose loss of connectivity might have a significant effect on brain function. Focusing on genes with more than one upstream effector and at least one downstream gene, we search for differential regulatory patterns by calculating the *exclusive effect* that is the effect of the mediator on its downstream gene after adjusting for its upstream genes. Non-significant exclusive effect corresponds to the loss of mediator.

We next assess the impact of the core on the transcription of other genes within each module, through the linear prediction model, and compare SCZ-cases versus controls. The cores that are good predictors for the transcription of downstream genes in both cases and controls are considered to be essential for the normal brain function.

By focusing on identified modules, we find new genes putatively related to SCZ using penalized linear regression and conditional analysis given the list of SCZ-associated genes mapped on the network at Step-2.

#### Step-5: Functional annotation and gene interactions

We look at different databases: the UniProt KnowledgeBase [19] for basic functional annotation and the proteins coded for the various genes; the Human Protein Atlas [20] and the Entrez Genes resource [21] for the level of expression in the human brain and other relevant tissues; and STRING database [22] for possible protein–protein interactions at different source of evidence. In addition, we perform over-representation analysis (i.e. “enrichment analysis”) on our set of genes newly associated with SCZ, using 4 different databases, KEGG pathways [23], Gene Ontology (GO) [24], Disease Ontology (DO) (https://www.disease-ontology.org), and Reactome Pathway Database (https://reactome.org) to investigate their potential biological functions.

#### Step-6: Replication of the pairwise relationship in the network

Using linear regression over the Allan Brain atlas data, we estimate the level of association between pairs of the genes in the networks and set the *p*-values cut off to 0.05 after adjusting for multiple testing based on false discovery rate (FDR).

## Results

We focused on one of the co-expression modules in [14] with 1181 transcripts, the module that includes the highest number of SCZ-associated genes, 104 SCZ-associated genes out of 1181. To have a mechanistic understanding of transcriptomic, we went beyond one-gene-at-a-time analysis and systematically integrated genetic, transcriptomic, and Hi-C data.

### Transcriptomic-causal network

We investigated interconnectivities among individual genes by utilizing principles of Mendelian randomization. To implement Mendelian randomization through instrumental variable technique and satisfy the assumptions, for each gene, we considered SNPs in 40 kb upstream/downstream of transcription start/end site so that we included variants in genes, promoters, and other proximal *cis*-regulatory elements. Furthermore, we used Hi-C data [17] for reflecting the secondary structure properties of looped DNA within a nucleus and selected the SNPs in the regions of the genome that interact with a gene. Therefore, the selected SNPs allowed us to consider genetic variants that mediate the effects of *cis*-regulatory elements via both short- and long-range interactions. In each region, we extracted the information/variation and generated new genetic variables. For details, see the method section. In total 4,641 variables were generated as potential IVs to identify causal relationships over 1181 transcripts and construct the transcriptomic-causal network.

### Permutation

We performed a permutation analysis to examine the stability of the network. The sets of randomly selected genes for permutation are entirely from the receptor (Fig. 2c, red curve) or broadcaster genes (Fig. 2c, blue curve) since the impact of receptors and broadcaster genes are different in the network. For each permutation, we selected 10 genes that all have the same number of out-/in-degree. The stability of the identified connections is very high (above 96%), even for the case of permuting the broadcaster or receptor genes with out-degree or in-degree ≥ 4. The results also provided supporting evidence for identified directions when permuted broadcaster genes (Fig. 2c, blue curve) showed more influence on the connections in the network in comparison with the receptor genes (Fig. 2c, red curve).

### Mapping the SCZ-associated genes on the network

To extract information from the transcriptomic-causal network regarding SCZ, we mapped SCZ-associated genes on the network. In Fig. 2a, blue colored nodes are SCZ-associated genes reported in previous studies (Additional file 1: Table 1). Mapping these genes on the network revealed underlying relationships among the genes. Figure 2e groups the SCZ-associated genes based on the type of studies that previously found the association (Additional file 1: Table 1). We measured the network properties and classified the genes as receptors, broadcasters, or mediators summarized in Fig. 2d for SCZ-associated genes. Looking at the impact of SCZ-associated genes on the network based on their out-degrees, we observed that less than 10% of the SCZ-associated genes have high impact (out-degree ≥ 4). This result can be an explanation for the small effect of most SCZ-associated genes on the disease processes.

**Table 1:** Novel SCZ associated genes and essential genes for brain functioning identified through regression and conditional analysis

Hypotheses			
Novel SCZ-associated genes		Essential genes for brain function	
Genes	Modules	Essential gene	SCZ-associated genes controlled by essential genes
*SEZ6L, RNF150*	*TENM3*-Module	*TENM3*	*BTRC, RNF150, SEZ6L, GRIN3A, VAT1L, GRIA1, XKR4*
*UNC5D*	*NRXN3*-Module	*NRXN3*	*CNTNAP2, ARFGEF3*
*RTF1*	*MYH10*-Module	*MYH10 *	*ANK2, LMO7, LRRC4C, DLG2, RTF1, PTPRK*
*FBXO32*	*PEX5L*-Module	*PEX5L*	*DLG2, STARD13*

Furthermore, we measured out-degree and in-degree for each gene in the network and investigated their relationship with the number of genetic variants that affected the genes. In the network built by integrating genetic variants and 1,181 gene transcriptions, 71 genetic variants showed significant effects on gene transcription levels. The distributions of degrees (the sum of in- and out-degrees) for the genes influenced and for those not influenced by any genetic variants are depicted in Fig. 2b. Both histograms showed a similar distribution pattern however the genes without the impact of any genetic variants have higher degree of connectivity. Therefore, we conjectured that transcriptions level of genes with high connectivity (degree ≥ 3) are less likely (OR = 0.5) to be influenced by the genetic variants (*cis*-regulation), thus their transcription is rather regulated directly or indirectly by other genes, mostly from different chromosomes (*trans-*regulatory factors) (Additional file 2: Figure  1A and Aditional file 1: Table 2 lists the genes influenced by genetic variants, together with in/out-degrees).

**Table 2:** Results of overrepresentation analysis for the enriched Novel SCZ associated genes

Enrichment	Pathway description	*p*-value	*p*. adjust	Gene symbol
KEGG	FoxO signaling pathway	0.0324	0.0445	*FBXO32*
KEGG	Axon guidance	0.0446	0.0446	*UNC5D*
GO	protein phosphorylated amino acid binding	0.0115	0.0319	*RTF1*
GO	RNA polymerase II complex binding	0.0119	0.0319	*RTF1*
GO	RNA polymerase core enzyme binding	0.0137	0.0319	*RTF1*
GO	basal transcription machinery binding	0.0162	0.0319	*RTF1*
GO	basal RNA polymerase II transcription machinery binding	0.0163	0.0319	*RTF1*
GO	RNA polymerase binding	0.0171	0.0319	*RTF1*
GO	phosphoprotein binding	0.0186	0.0319	*RTF1*
GO	single-stranded DNA binding	0.0253	0.0380	*RTF1*
Reactome	FOXO-mediated transcription of oxidative stress, metabolic and neuronal genes	0.0081	0.0323	*FBXO32*
Reactome	Netrin-1 signaling	0.0140	0.0323	*UNC5D*
Reactome	E3 ubiquitin ligases ubiquitinate target proteins	0.0165	0.0323	*RTF1*
Reactome	Formation of RNA Pol II elongation complex	0.0171	0.0323	*RTF1*
Reactome	RNA Polymerase II Transcription Elongation	0.0171	0.0323	*RTF1*
Reactome	FOXO-mediated transcription	0.0182	0.0323	*FBXO32*
Reactome	Protein ubiquitination	0.0221	0.0323	*RTF1*
Reactome	RNA Polymerase II Pre-transcription Events	0.0234	0.0323	*RTF1*
DO	lateral sclerosis	0.0275	0.0813	*FBXO32*
DO	amyotrophic lateral sclerosis	0.0383	0.0813	*FBXO32*
DO	motor neuron disease	0.0462	0.0813	*FBXO32*
DO	chronic obstructive pulmonary disease	0.0542	0.0813	*FBXO32*
DO	obstructive lung disease	0.0755	0.0905	*FBXO32*

We estimated the association of 1653 pairs of genes linked in the identified network from the replication set. Although, the sample size in the replication dataset was small, 65% of the pairwise relationships were significant at 0.05 *p*-value cut off after FDR adjustment (Additional file 2: Figure 1B).

### Differential gene regulatory patterns

**Fig. 3:**
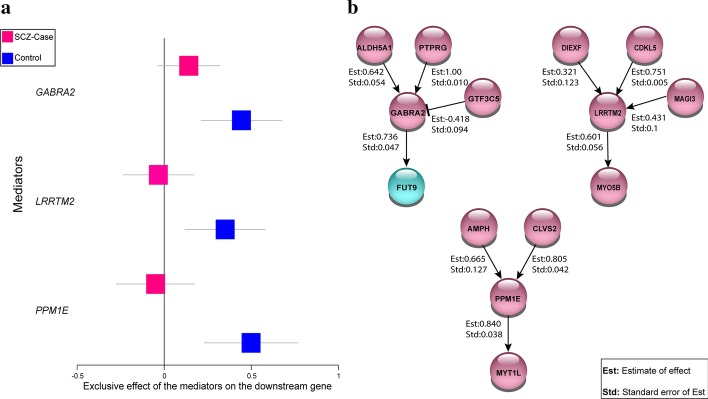
**a** Effect of the mediators exclusively expressed in brain on the downstream genes. The forest plot represents 95% confidence intervals of the effect size in downstream gene transcription by the different mediator gene, red: mean effect size for SCZ-cases, blue: mean effect size for controls. Non-significant effect corresponds to loss of mediators. **b** Upstream genes and downstream genes for the mediators (*GABRA2, LRRTM2, PPM1E*) and their causal effects in controls. The arrows represent transcription activation (positive effect size). The line with capped end represents transcriptional repression (negative effect size). In cyan is the gene reported as SCZ-associated gene in previous studies. For all interactions, the estimate of the effect size (Est) and standard deviation (Std) are shown

To identify differentially gene regulatory pattern, we focused on the loss of mediators among the genes with more than one upstream effector. We identified 9 mediator genes lost their interactions with downstream genes in SCZ compared to controls. Among these, 3 genes (*GABRA2*, *LRRTM2, PPM1E)* are primarily expressed in human brain tissue (Additional file 1: Table 3) (https://www.proteinatlas.org). Figure 3a shows the exclusive effect of these three genes on their downstream genes in the network and represents that the effects on downstream genes are not significant for SCZ-cases corresponded to loss of mediators. The subnetworks of these mediators depicted in Fig. 3b show the causal effect size of each transcription level on the downstream gene in controls.

Importantly, based on the STRING database [22], these interactions are validated at the protein level. More specifically, the corresponding proteins GABRA2, LRRTM2, and PPM1E interact indirectly with the proteins encoded by the downstream genes in our network (Additional file 1: Table 4) [19]. Therefore, these proteins may be important for the normal brain function since their corresponding genes (mediators) lost their effect on the downstream gene transcriptional regulation in SCZ-cases.

**Fig. 4:**
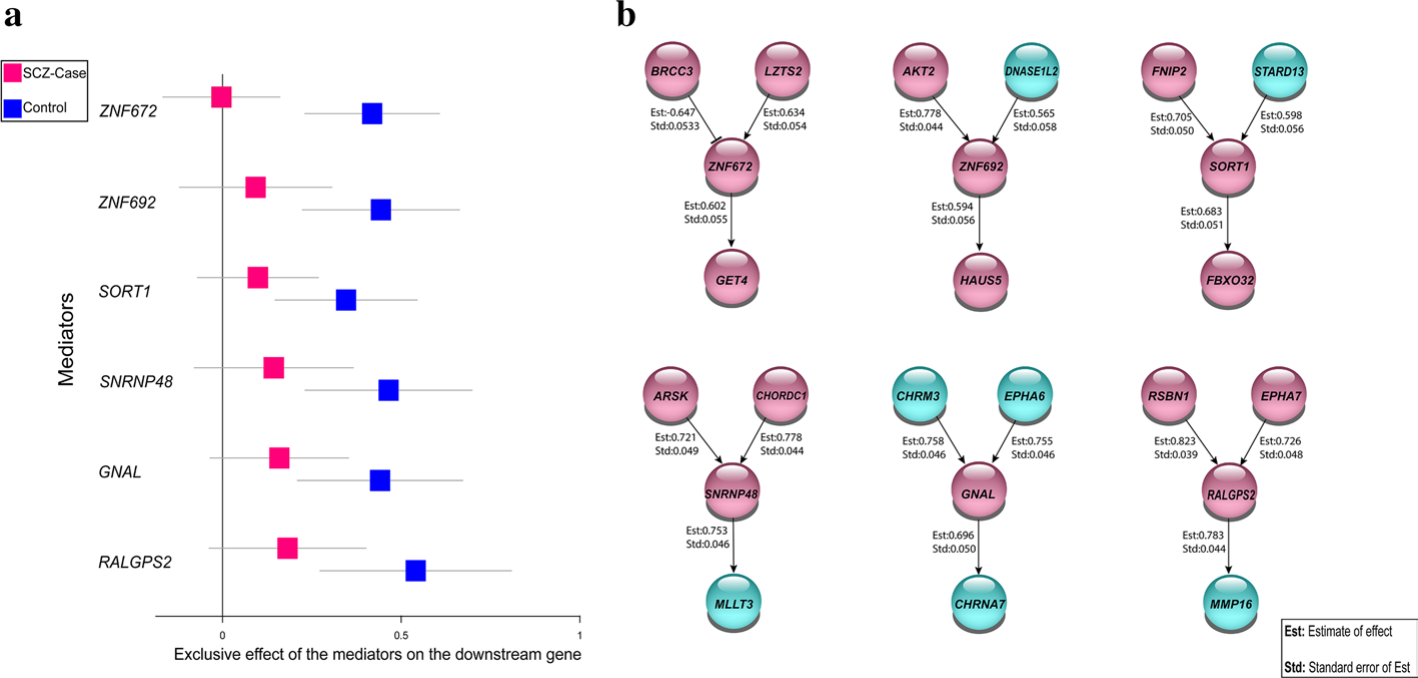
**a** Effect of the mediator genes essential for normal brain functioning on the downstream genes. The forest plot represents 95% confidence intervals of the effect size in downstream gene transcription by the different mediator gene, red: mean effect size for SCZ-cases, blue: mean effect size for controls. Non-significant effect corresponds to loss of mediators. **b** Upstream genes and downstream genes for the mediators (*ZNF672, ZNF692, SORT1, SNRNP48, GNAL, RALGPS2*) and their causal effects. The arrows represent transcription activation (positive effect size). The line with capped end represents transcriptional repression (negative effect size). In cyan there are the genes reported as associated with SCZ in previous studies. For all interactions, the effect size (Est) and standard deviation (Std) are shown

Among the other genes with loss of mediators (Fig. 4), *SORT1* and *GNAL* have the highest expression in the brain in comparison to other human tissues according to HPA [20]; *ZNF692* and *RALGPS2* have the highest expression in the testis; *ZNF672* and *SNRNP48* are expressed in all tissues including the brain (Additional file 1: Table 5) (https://www.proteinatlas.org). Based on the STRING database [22], the corresponding proteins produced by all these genes interact directly/indirectly with the proteins encoded by the downstream genes in the network over the control group (Additional file 1: Table 6). These genes (mediators) lost their impact on the downstream gene transcriptional regulation in SCZ-cases; therefore, we hypothesized these genes are implicated in SCZ.

### Novel genes putatively related to SCZ and essential genes for the brain function

Identifying modules facilitates the discovery of essential genes for brain function and identifying novel genes related to SCZ. We predicted the transcription level of the downstream genes in the identified modules (Additional file 3: Modules) using the transcription level of the corresponding core in both groups, SCZ-cases and controls. The core that is good predictor for its downstream genes in both groups are hypothesized as essential genes for brain functioning. Moreover, we discovered new genes putatively related to SCZ (Table 1) using the predicting model and conditional analysis. We performed enrichment analysis on the set of identified novel genes related to SCZ, using 4 different databases, KEGG pathways [23], Gene Ontology (GO) [24], Disease Ontology (DO) (https://www.disease-ontology.org), and Reactome Pathway Database (https://reactome.org). After adjusting *p*-values based on FDR correction, we selected genes with an adjusted *p*-value at level 0.05 for at least three of the databases (GO, KEGG, DO, or Reactome). Table 2 shows that *UNC5D*, *FBXO32,* and *RTF1* are enriched and have potential biological functions related to SCZ.

### Other properties

**Fig. 5:**
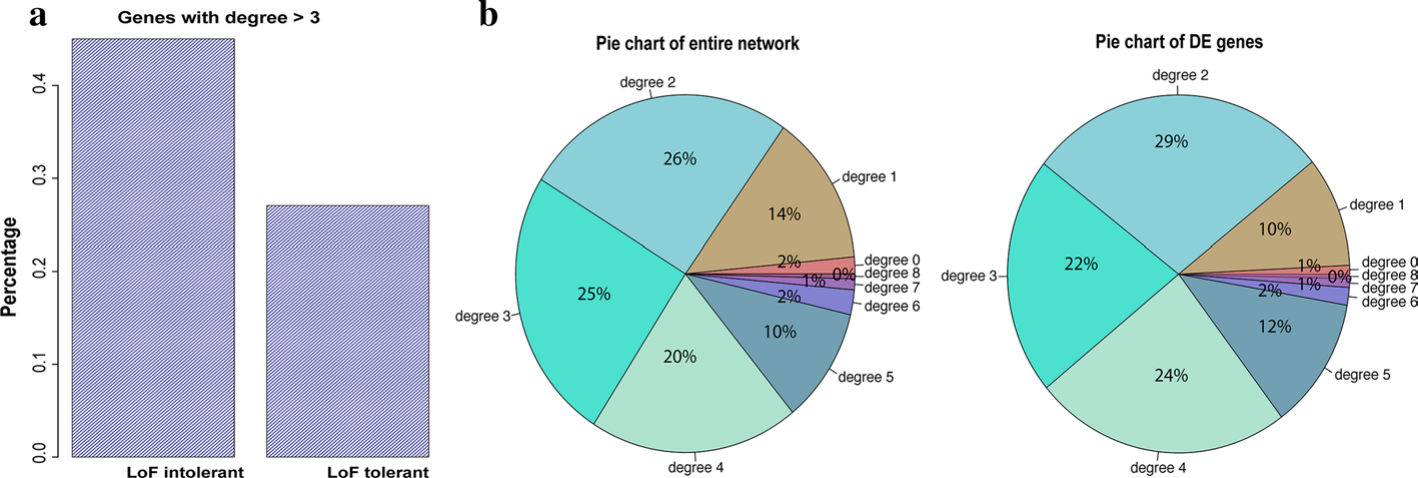
**a** Histogram of high degree of connectivity (degree > 3) across LoF intolerant and LoF tolerant genes in the network. **b** Pie chart of frequency of genes regarding the degree of connectivity for entire network and for subset of differentially expressed genes

We finally investigated the properties of loss-of-function mutation (LoF) intolerant and LoF tolerant genes in the network. We observed that 45% of the LoF intolerant genes have a high degree of connectivity (degree ≥ 4) which dropped to 27% for LoF tolerant genes (Fig. 5a). Therefore, probability of loss-of-function intolerant was positively correlated with the connectivity of the genes in the network similar to what Lek et al. [25] observed. In the network, we then explored the relationship of differentially expressed genes published by Hauberg et al. [2] with the degree of connectivity and found no difference as compared to the entire network (Fig. 5b).

## Discussion

Despite transformative advances in technology, it remains difficult to assess where we are in understanding of a complete comprehension of the biological mechanisms [9]. One of the difficulties is that the research tools seldom provide insights into aspects of the overall picture of the system. The focus of the majority of analytical tools in use is still on a single dimension of data, rather than integrating data across different dimensions simultaneously/systematically to view processes more completely. While the technology to measure different levels of biology in large scale is being developed at high pace, the algorithms and analytical methods do not progress at the same rate, and hence new algorithms and intellectual approaches must be devised to take advantage of the availability of such wealth of data.

In this study, we systematically integrated genotype, Hi-C, and RNA-seq data by using a novel causal network reconstruction algorithm and focused on understanding gene transcription regulation in the human brain in the context of SCZ. The integration was based on our observations that the transcription of a gene may be affected by genetic variants in cis-regulatory regions, but it can also be mediated by transcribed distal genes (trans-regulatory factors). We demonstrated that the properties of causal networks provide tools to improve our understanding of the brain functioning mechanism as well as tools for comparing the gene regulatory pattern between SCZ-cases and controls. We replicated the relation between genes identified based on data from the CMC consortium using the RNA-seq dataset from the Allen Brain atlas. We showed that our network predictions either recapitulate known biology or can be prospectively validated, demonstrating a high degree of accuracy in the predicted network.

Through investigating the network properties, we observed that genes with fewer connections are more likely to be affected by genetic variants than those highly connected in the network, but the latter is more often found regulated by or regulating other distal genes as *trans-*regulatory factors. We concluded that genetic variation in the context of human brain tissue works as *cis*-regulatory elements for genes that are not so much involved in *trans*-regulatory interactions. This property may be general for gene regulatory networks across the tissues. In the network, we observed less than 10% of SCZ-associated genes previously reported are high impact genes (out-degree $\ge$ 4). This result can be an explanation for the small effect of most SCZ-associated genes on the disease processes.

Our systematic-integrative approach elucidates the regulatory context for essential genes in the brain. Besides, the proposed approach is also able to identify the genes that are differentially regulated in SCZ-cases. Altogether this work presents a significant step towards the systematic understanding of the genetic mechanisms of schizophrenia and paves the way for designing new and more selective treatments that minimize undesired side effects. For instance, one of the identified differential regulated genes, *GNAL,* is a mediator between two SCZ drug targets (*CHRM3* and CHRNA7). The protein coded by the upstream gene, *CHRM3,* is targeted by antipsychotic drugs against schizophrenia and bipolar disorder [26]. *CHRNA7* downstream gene of *GNAL* is also considered a promising drug target for the treatment of cognitive dysfunction in schizophrenia and improves memory and executive functions in patients and healthy individuals. However, clinical trials with pro-cognitive drugs are challenged by large inter-individual response variations [27]. Identification of this differential regulatory pattern suggests that *GNAL* can be an alternative SCZ drug target since it encodes a G protein alpha subunit widely expressed in the central nervous system.

The novel identified SCZ associated genes *UNC5D*, *FBXO32,* and *RTF1* are enriched in pathways related to neural development. *UNC5D* is enriched in the ‘axon guidance pathway’ and Netrin-1 (a protein required during axon guidance) signaling according to the KEGG pathway database. Interestingly, axon guidance impacts how white matter tract is formed by pre-target axon order in normal development, whereas abnormalities in white matter tract have been early reported in SCZ [28]. *RTF1* is enriched in protein ubiquitination as divided by Reactome pathway databases. Protein ubiquitination has been known to be of key importance in neural development and at maintenance of brain structure and function. In SCZ, the ubiquitin–proteasome system is dysregulated [29]. Moreover, recently, in a pre-print, the proteasome dysfunction has been related to aggregation of ubiquitinated proteins [30]. An enrichment for sclerosis-related as well as pulmonary disease is found in *FBXO32*, following the DO database. Not surprisingly, according to the KEGG database, *FBXO32* is enriched in the *FOXO* genes signaling pathway, which is present in many important cellular processes such as cell cycle, apoptosis, metabolism, oxidative stress, immune regulation, etc. [31, 32].

The utilities of the transcriptomic-causal network are beyond single gene analysis, such as TWAS (see [33] and references therein) or differential expression analysis [34]. For instance, investigating different regulatory patterns between cases and controls is possible through the transcriptomic-causal network but not with differential expression analysis or TWAS. The transcriptomic-causal network discovers the key drivers and represents the effect of a specific gene on the transcriptomic system, and therefore, provides possibility to better design experiments in future studies that cannot be elucidated by single gene analysis. The transcriptomic network analysis is a systematic analysis of genes (study of a set of genes simultaneously) regardless of being expressed by GWAS loci or altered by environmental factors and uses genetic information as a tool to identify causal relationships among genes based on the principle of Mendelian randomization/instrumental variables. However, some studies such as TWAS aim to prioritize genes at GWAS loci [33], i.e., aim to find pathways from genetic variants to disease via genes through one-gene-at-a-time analysis.

## Conclusions

Overall, the transcriptomic-causal network analysis which integrates genotypes, Hi-C and transcriptomic data systematically is complementary to experimental research to unveil the mechanisms that control the dysregulated gene transcription patterns in SCZ. The integrative systems approaches will allow the design of further experiments to target the relevant genes, either by editing them or drug targeting, without disrupting essential pathways for normal brain function and as a result minimizing undesired side effects.

## Method

### An overview on application of Mendelian randomization/instrumental variable technique

To gain sufficient understanding and predict the behavior of a system, Mendelian randomization technique that uses genetic variants as instrumental variables has recently gained attention. Instrumental variables control better for unmeasured confounders relative to other approaches such as regression, matching, and propensity score methods [35–37]. This feature leads to robust results with mechanistic understanding (i.e. causal interpretation) even in the presence of unmeasured confounders and reverse causations if the utilized genetic variants qualify as instrumental variables:

1. Being robustly associated with a transcript of interest (exposure) that is a potential cause of an outcome transcript.
2. Not being associated with any confounders (measured and unmeasured) of the two transcripts of interest
3. Not being associated with the transcript outcome except through the transcript of interest.

**Fig. 6:**
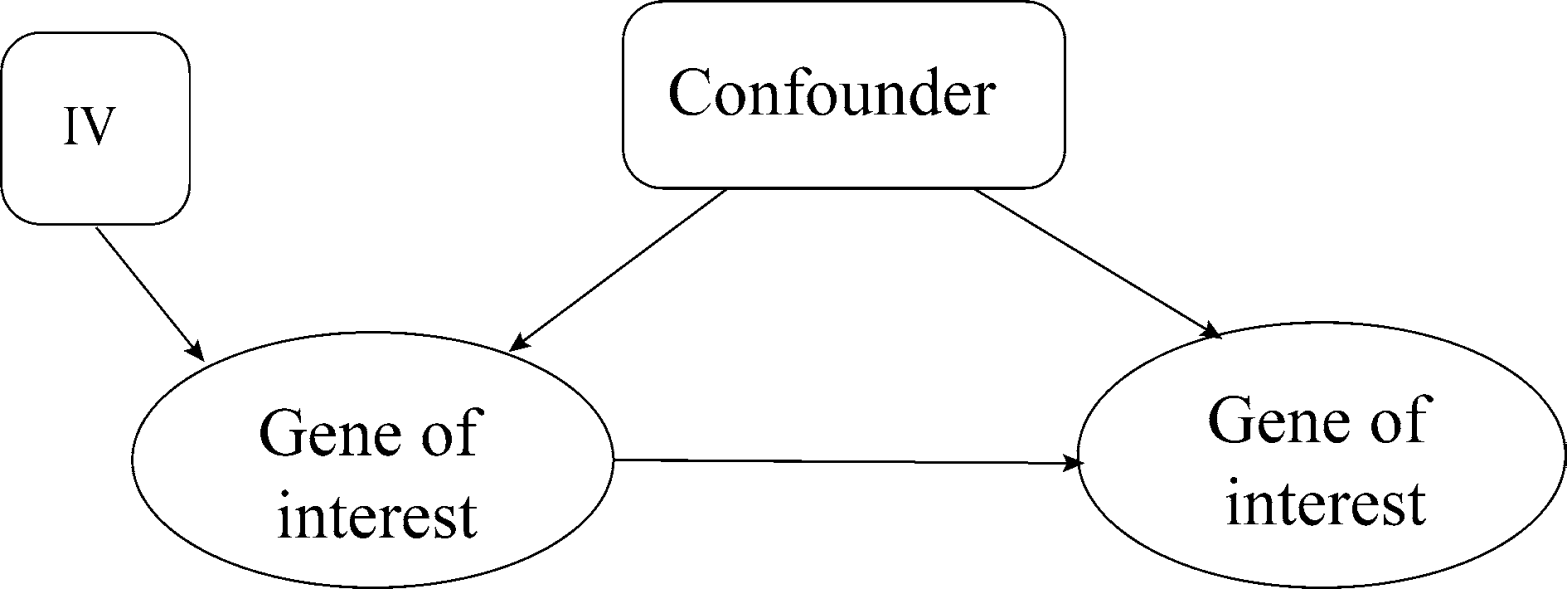
Schematic visualization of instrumental variable assumptions. IV stands for instrumental variable

These conditions are visualized in Fig. 6 on a small scale.

**Fig. 7:**
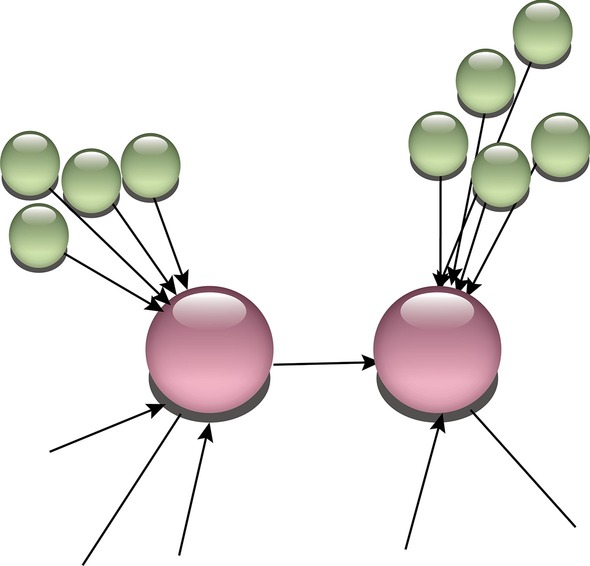
Visualization 3 of main features of generated instrumental variables from multiple SNPs using exome and Hi-C data. The aim is to identify underlying relationship between the transcripts (Pink) using the instrumental variables (green)

Genotypic features such as pleiotropy, the presence of linkage disequilibrium, genetic heterogeneity, lack of knowledge about the confounders, and population stratification; may violate the assumptions of instrumental variables and bring limitations to the application of genetic variants [36]. These assumptions must be justified by background knowledge of the underlying biology. However, evaluating the assumptions adds to the credibility of the analyses, and there are some approaches toward this end. Some of the approaches aim to find a single genetic variant strongly correlated with the variable of interest. These approaches are limited to identify sufficient numbers of instrumental variables for a large-scale data set [38] and possibility violate some of the assumptions due to the genotype features such as the pleiotropic effect. Our approach aims to hold the underlying assumptions through three main features as the following (Fig. 7):

1. Extracting information from several single nucleotide polymorphism (SNP)/genes to create stronger genetic variables than a single SNP/gene.
2. Generating independent variables and possibility of allocating multiple instrumental variables to a transcript to explain the variation of the transcript sufficiently.
3. Selecting IVs after assessing the independency of IVs and the outcome given the exposure to avoid violation of the assumptions, due to genetic variants’ properties such as pleiotropic effect.

These features of the algorithm account for the variation in transcripts that are free of confounding.

### Generating instrumental variables

To investigate interconnectivity among transcripts and construct a transcriptomic-causal network, we apply Bayesian networks and instrumental variables [39]. The challenge is finding IVs that satisfy the assumptions. The two approaches that find SNPs associated to genes strongly as IVs [39] or generating strong IVs through extracting information from entire genome [37] found almost no IVs. The result is consistent with the fact that gene expression is strictly controlled by the interplay of regulatory events at gene promoters and *trans-*regulatory elements. Therefore, we conclude that gene transcription is affected by genetic variations nearby (orientation, position, and distance). This result is also reported recently by [40]. To generate the IVs based on genetic variants nearby genes, we include variants in genes and their promoters as well as the variants that are reflecting the secondary structure properties of looped DNA within a nucleus. Therefore, we select the genetic variants in the regions of the genome that interact with a particular gene based on genotype and Hi-C data with the aim of considering genetic variants that mediate the effects of *cis*-regulatory elements via both short- and long-range interactions.

To generate IVs and fulfill the underlying aforementined assumptions, we apply multiple correspondence analysis [41], which is a generalization of principal component analysis for categorical variables, over a set of variants nearby each gene, as defined above. Denoting genetic variants as (X_1_,X_2_,…,X_n_) while each X_i_ represents an SNP with 3 categories {0,1,2}, we define one indicator variable for each category and scale the new matrix for its grand total and name it **Y**. We then obtain factor scores based on the following singular value decomposition,

$${\mathbf{R}}^{{ - \frac{1}{2}}} \left( {{\mathbf{Y}} - {\text{rc}}} \right){\mathbf{C}}^{{ - \frac{1}{2}}} = {\mathbf{F\Lambda }}^{\frac{1}{2}} {\mathbf{T}}^{{\text{t}}}$$

where ${{\varvec{\Lambda}}}$ is the diagonal matrix of eigenvalues, **R** = diag{r}, **C** = diag{c}, **r** and **c** are the vector of row total and column total of **Y** respectively.

### Transcriptomic-causal network

A causal network is a Bayesian network augmented with Mendelian randomization principles implemented with IV techniques. The Bayesian network of *p* genes and genetic variants $\left( {g_{1} , g_{2} , \ldots ,g_{p} } \right)$ can be represented based on their joint probability as

$$f\left( {g_{1} , g_{2} , \ldots , g_{p} } \right) = \mathop \prod \limits_{i} f\left( {g_{i} |S_{{g_{i} }} } \right)$$

where $S_{{g_{i} }}$ is the set of upstream genes for *i*th gene and $f\left( {g_{1} , g_{2} , \ldots , g_{p} } \right)\sim N\left( {0, {\Sigma }} \right)$. The stability of the identified Bayesian networks in an observational study is established in Mendelian principles since the genetic inherited variation is the cause of phenotypic variation (here gene transcription) [42]. These stable Bayesian networks are called causal networks in observational studies [8, 36, 37] which is compatible with the structural equation model.

For quality of the fit, we employ Hamming distance [18] for different tuning parameter {0.01, 0.005, 0.001, 0.0005} and selected 0.001 as the tuning parameter that minimizes the average of Hamming distance. Furthermore, we design a permutation analysis to test the stability of the network and directionality. We repeat this permutation for different degrees of connectivity {1,…,5} separately and calculate the number of connectivity that changes in comparison with the identified network without permutation.

### Differential gene regulation pattern

We use structural equation modeling to estimate the causal effect of each gene, $Z_{i}$, for SCZ-cases and control group as

$$Z = \left( {I - {\Lambda }} \right)U, U^{T} \sim N_{p} \left( {0, \Delta } \right)$$

where $Z = \left\{ {Z_{1} ,Z_{2} , \ldots , Z_{p} } \right\}, \quad \Delta = diag\left\{ {\sigma_{i}^{2} } \right\}, {\Lambda }$ is lower diagonal matrix, corresponding to the effect of transcriptomics on each other. The non-zero entries are determined from the network in Fig. 2a to satisfy the assumptions of structural equation.

For the set of interaction with significant causal effect, we calculate the exclusive effect of each gene on its downstream genes and test if they are significant based on 95% confidence interval. Comparing the significant ones in controls and cases, we aim to find a set of mediators that lost their interaction with their downstream in cases.

### Predicting gene transcription level

Further analysis of transcriptomic-causal network narrows down the search space for identifying predictors for the transcript of the target gene (*g*). A subset of genes (*S*) that directly interact with *g* are the best predictors of *g*. Therefore, using the causal network, we select the best predictors and fit a regression model to predict the transcription level of *g*. Since the predictors may be correlated, we impose penalized term of norm 2 to our model such that the loss function of the model is:

$$\frac{1}{2}\left\| {{\text{g}} - {\text{S}}\beta } \right\|_{2}^{2} + \nu \left\| \beta \right\|_{1}^{2},$$

where $\nu$ is the tuning parameter. To assess prediction performance, we use the cross-validation technique. We train the penalized regression model using four-fifth of control set data and test the prediction performance of the model using left-out data. We calculate the correlation between predicted and observed values as well as square predicted error. We repeat 200 times the entire procedure of fivefold cross-validation and report the average of correlation and mean square predicted error (MSPE) [43] and select the genes with the correlation above 0.6 and MSPE less than 0.3 as good predictors. In this step, the transcriptions are scaled for having unit standard deviations and producing comparable results.

### Enrichment analysis

In this study, we use enrichment analyses such as GO based on hypergeometric distribution. The analyses aim to determine whether any terms annotate a list of the gene at frequency greater than that expected by chance when

$$p = 1 - \mathop \sum \limits_{i = 0}^{k - 1} \frac{{\left( {\begin{array}{*{20}c} {N - M} \\ {n - i} \\ \end{array} } \right)\left( {\begin{array}{*{20}c} M \\ i \\ \end{array} } \right)}}{{\left( {\begin{array}{*{20}c} N \\ n \\ \end{array} } \right)}},$$

where *N* and *M* represent the total number of genes in the background and the number of genes within that distribution annotated to the terms of interest, respectively; *n* and *k* are the size of the list of significant gene and the number of genes within that list which are annotated to the term, respectively [44]. Then, the calculated *p*-values are adjusted for multiple comparisons based on false discovery rate.

## Supplementary information

**Additional file 1:** **Table 1.** Previously identified SCZ-associated genes with in/outdegree in this study. **Table 2.** List of the genes influenced by genetic variants or IVs together with in/out-degrees. **Table 3.** Loss of mediator genes with biased expression in the human brain and their upstream and downstream genes in the network. **Table 4.** Interactions of the proteins encoded by the loss of mediator genes and upstream and downstream genes in Table 3. **Table 5.** Genes with loss of mediator expressed in different human tissues together with their downstream and upstream genes. **Table 6.** Interactions of the proteins encoded by the loss of mediator genes and their upstream and downstream genes in Table 5.

**Additional file 2:** **Figure 1.** **A:** Circos plot represents the interaction of the genes from different chromosomes; **B:** Pie-chart of the replication analysis after FDR adjustment.

**Additional file 3:** Modules.

## Data Availability

The Genotype and RNA-seq data are available in the CommonMind Consortium (CMC; https://www.synapse.org/CMC). Hi-C data can be downloaded from supplementary tables 22 and 23 in the study by Won et al. [17].

## Abbreviations

CMC: CommonMind consortium
FDR: False discovery rate
HPA: Human protein atlas
IV: Instrumental variable
LoF: Loss-of-function
MSPE: Mean square predictive error
SNP: Single nucleotide polymorphism
SCZ: Schizophrenia
