# Supplementary material for: Differential gene regulatory pattern in the human brain from schizophrenia using transcriptomic-causal network
Source: BMC Bioinformatics. 2020 Oct 21;21:469. doi: 10.1186/s12859-020-03753-6 (PMC7579819; doi:10.1186/s12859-020-03753-6)
Supplement: Supplementary file 1 — Additional file 1: Table 1. Previously identified SCZ-associated genes with in/outdegree in this study. Table 2. List of the genes influenced by genetic variants or IVs together with in/out-degrees. Table 3. Loss of mediator genes with biased expression in the human brain and their upstream and downstream genes in the network. Table 4. Interactions of the proteins encoded by the loss of mediator genes and upstream and downstream genes in Table 3. Table 5. Genes with loss of mediator expressed in different human tissues together with their downstream and upstream genes. Table 6. Interactions of the proteins encoded by the loss of mediator genes and their upstream and downstream genes in Table 5. [file 12859_2020_3753_MOESM1_ESM.docx]

**Differential gene regulatory pattern in the human brain from schizophrenia using transcriptomic-causal network**

Akram Yazdani*, Raul Mendez-Giraldez, Azam Yazdani, Michael R Kosorok, Panos Roussos*

**Additional file 1: Tables**

**Table 1:** Previously identified SCZ-associated gene with in/out-degree in this study

| MAPPED  Gene | Gene | scz-denovo-nonsyn | scz-gwas | psych-cnv | scz-denovo-lof | in-degree | out-degree |
| --- | --- | --- | --- | --- | --- | --- | --- |
| *ACACA* | ENSG00000132142 |  |  | psych-cnv |  | 1 | 1 |
| *CHRNA7* | ENSG00000175344 |  |  | psych-cnv |  | 3 | 0 |
| *COMT* | ENSG00000093010 |  |  | psych-cnv |  | 1 | 4 |
| *CRKL* | ENSG00000099942 |  |  | psych-cnv |  | 0 | 0 |
| *FMN1* | ENSG00000248905 |  |  | psych-cnv |  | 3 | 0 |
| *GABRB3* | ENSG00000166206 |  |  | psych-cnv |  | 3 | 2 |
| *KIAA0430* | ENSG00000166783 |  |  | psych-cnv |  | 3 | 1 |
| *MYO19* | ENSG00000141140 |  |  | psych-cnv |  | 3 | 1 |
| *UBXN7* | ENSG00000163960 |  |  | psych-cnv |  | 4 | 0 |
| *CCDC39* | ENSG00000145075 |  |  |  | scz-denovo-lof | 3 | 1 |
| *CNTN3* | ENSG00000113805 |  |  |  | scz-denovo-lof | 1 | 1 |
| *DLG2* | ENSG00000150672 |  |  |  | scz-denovo-lof | 2 | 0 |
| *ITSN1* | ENSG00000205726 |  |  |  | scz-denovo-lof | 4 | 0 |
| *KIAA1429* | ENSG00000164944 |  |  |  | scz-denovo-lof | 2 | 0 |
| *KPNA1* | ENSG00000114030 |  |  |  | scz-denovo-lof | 0 | 3 |
| *PAQR9* | ENSG00000188582 |  |  |  | scz-denovo-lof | 3 | 0 |
| *TLK1* | ENSG00000198586 |  |  |  | scz-denovo-lof | 1 | 1 |
| *ZEB1* | ENSG00000148516 |  |  |  | scz-denovo-lof | 1 | 1 |
| *ZMYND11* | ENSG00000015171 |  |  |  | scz-denovo-lof | 1 | 5 |
| *ALDH5A1* | ENSG00000112294 | scz-denovo-nonsyn |  |  |  | 0 | 2 |
| *ARRDC1* | ENSG00000197070 | scz-denovo-nonsyn |  |  |  | 1 | 3 |
| *C14orf28* | ENSG00000179476 | scz-denovo-nonsyn |  |  |  | 1 | 1 |
| *C9orf114* | ENSG00000198917 | scz-denovo-nonsyn |  |  |  | 1 | 2 |
| *CBR4* | ENSG00000145439 | scz-denovo-nonsyn |  |  |  | 1 | 2 |
| *CCDC39* | ENSG00000145075 | scz-denovo-nonsyn |  |  |  | 3 | 1 |
| *CNTN3* | ENSG00000113805 | scz-denovo-nonsyn |  |  |  | 1 | 1 |
| *CUL3* | ENSG00000036257 | scz-denovo-nonsyn |  |  |  | 0 | 3 |
| *DLG2* | ENSG00000150672 | scz-denovo-nonsyn |  |  |  | 2 | 0 |
| *DNASE1L2* | ENSG00000167968 | scz-denovo-nonsyn |  |  |  | 1 | 1 |
| *FAM160B1* | ENSG00000151553 | scz-denovo-nonsyn |  |  |  | 5 | 2 |
| *FSD1L* | ENSG00000106701 | scz-denovo-nonsyn |  |  |  | 2 | 1 |
| *GFOD1* | ENSG00000145990 | scz-denovo-nonsyn |  |  |  | 0 | 2 |
| *GRIN2A* | ENSG00000183454 | scz-denovo-nonsyn |  |  |  | 3 | 3 |
| *ITM2B* | ENSG00000136156 | scz-denovo-nonsyn |  |  |  | 0 | 2 |
| *ITSN1* | ENSG00000205726 | scz-denovo-nonsyn |  |  |  | 4 | 0 |
| *KCNQ5* | ENSG00000185760 | scz-denovo-nonsyn |  |  |  | 2 | 1 |
| *KIAA1109* | ENSG00000138688 | scz-denovo-nonsyn |  |  |  | 2 | 2 |
| *KIAA1244* | ENSG00000112379 | scz-denovo-nonsyn |  |  |  | 2 | 0 |
| *KIAA1429* | ENSG00000164944 | scz-denovo-nonsyn |  |  |  | 2 | 0 |
| *KIAA2018* | ENSG00000176542 | scz-denovo-nonsyn |  |  |  | 2 | 3 |
| *KLF12* | ENSG00000118922 | scz-denovo-nonsyn |  |  |  | 4 | 1 |
| *KPNA1* | ENSG00000114030 | scz-denovo-nonsyn |  |  |  | 0 | 3 |
| *LUZP1* | ENSG00000169641 | scz-denovo-nonsyn |  |  |  | 4 | 0 |
| *MAMDC4* | ENSG00000177943 | scz-denovo-nonsyn |  |  |  | 0 | 1 |
| *MYH10* | ENSG00000133026 | scz-denovo-nonsyn |  |  |  | 2 | 3 |
| *PAQR9* | ENSG00000188582 | scz-denovo-nonsyn |  |  |  | 3 | 0 |
| *PBX1* | ENSG00000185630 | scz-denovo-nonsyn |  |  |  | 3 | 0 |
| *PIK3CB* | ENSG00000051382 | scz-denovo-nonsyn |  |  |  | 0 | 5 |
| *POLL* | ENSG00000166169 | scz-denovo-nonsyn |  |  |  | 3 | 0 |
| *POMT1* | ENSG00000130714 | scz-denovo-nonsyn |  |  |  | 2 | 1 |
| *PRUNE2* | ENSG00000106772 | scz-denovo-nonsyn |  |  |  | 1 | 3 |
| *PTPN3* | ENSG00000070159 | scz-denovo-nonsyn |  |  |  | 2 | 0 |
| *PTPRG* | ENSG00000144724 | scz-denovo-nonsyn |  |  |  | 3 | 1 |
| *RIF1* | ENSG00000080345 | scz-denovo-nonsyn |  |  |  | 2 | 3 |
| *SDF4* | ENSG00000078808 | scz-denovo-nonsyn |  |  |  | 0 | 4 |
| *SLC4A8* | ENSG00000050438 | scz-denovo-nonsyn |  |  |  | 1 | 1 |
| *SON* | ENSG00000159140 | scz-denovo-nonsyn |  |  |  | 3 | 0 |
| *STARD13* | ENSG00000133121 | scz-denovo-nonsyn |  |  |  | 2 | 1 |
| *STK38L* | ENSG00000211455 | scz-denovo-nonsyn |  |  |  | 0 | 2 |
| *SYNE1* | ENSG00000131018 | scz-denovo-nonsyn |  |  |  | 2 | 1 |
| *TLK1* | ENSG00000198586 | scz-denovo-nonsyn |  |  |  | 1 | 1 |
| *TNKS* | ENSG00000173273 | scz-denovo-nonsyn |  |  |  | 3 | 0 |
| *VPS13A* | ENSG00000197969 | scz-denovo-nonsyn |  |  |  | 5 | 0 |
| *XPO7* | ENSG00000130227 | scz-denovo-nonsyn |  |  |  | 0 | 1 |
| *ZEB1* | ENSG00000148516 | scz-denovo-nonsyn |  |  |  | 1 | 1 |
| *ZMYND11* | ENSG00000015171 | scz-denovo-nonsyn |  |  |  | 1 | 5 |
| *ZNF721* | ENSG00000182903 | scz-denovo-nonsyn |  |  |  | 3 | 0 |
| *BTRC* | ENSG00000166167 |  | scz-gwas |  |  | 4 | 0 |
| *CACNB2* | ENSG00000165995 |  | scz-gwas |  |  | 3 | 0 |
| *CAMTA1* | ENSG00000171735 |  | scz-gwas |  |  | 3 | 2 |
| *CCDC39* | ENSG00000145075 |  | scz-gwas |  |  | 3 | 1 |
| *CEP170* | ENSG00000143702 |  | scz-gwas |  |  | 1 | 3 |
| *CREB1* | ENSG00000118260 |  | scz-gwas |  |  | 1 | 3 |
| *CREBRF* | ENSG00000164463 |  | scz-gwas |  |  | 0 | 2 |
| *DACH1* | ENSG00000165659 |  | scz-gwas |  |  | 3 | 0 |
| *EYS* | ENSG00000188107 |  | scz-gwas |  |  | 2 | 2 |
| *FAM63B* | ENSG00000128923 |  | scz-gwas |  |  | 0 | 4 |
| *FUT9* | ENSG00000172461 |  | scz-gwas |  |  | 2 | 2 |
| *GRIA1* | ENSG00000155511 |  | scz-gwas |  |  | 2 | 3 |
| *GRIN2A* | ENSG00000183454 |  | scz-gwas |  |  | 3 | 3 |
| *IPO9* | ENSG00000198700 |  | scz-gwas |  |  | 2 | 0 |
| *KCND3* | ENSG00000171385 |  | scz-gwas |  |  | 3 | 1 |
| *KIAA1109* | ENSG00000138688 |  | scz-gwas |  |  | 2 | 2 |
| *ME1* | ENSG00000065833 |  | scz-gwas |  |  | 0 | 1 |
| *MFAP3* | ENSG00000037749 |  | scz-gwas |  |  | 1 | 1 |
| *MKLN1* | ENSG00000128585 |  | scz-gwas |  |  | 2 | 3 |
| *MMP16* | ENSG00000156103 |  | scz-gwas |  |  | 2 | 2 |
| *NAP1L1* | ENSG00000187109 |  | scz-gwas |  |  | 5 | 0 |
| *NR3C2* | ENSG00000151623 |  | scz-gwas |  |  | 0 | 1 |
| *NRXN3* | ENSG00000021645 |  | scz-gwas |  |  | 1 | 5 |
| *NTRK3* | ENSG00000140538 |  | scz-gwas |  |  | 1 | 2 |
| *OSBPL10* | ENSG00000144645 |  | scz-gwas |  |  | 1 | 1 |
| *PCNX* | ENSG00000100731 |  | scz-gwas |  |  | 1 | 3 |
| *PHLDA1* | ENSG00000139289 |  | scz-gwas |  |  | 3 | 1 |
| *PIAS2* | ENSG00000078043 |  | scz-gwas |  |  | 0 | 2 |
| *PTPRG* | ENSG00000144724 |  | scz-gwas |  |  | 3 | 1 |
| *SACS* | ENSG00000151835 |  | scz-gwas |  |  | 3 | 1 |
| *TENM3* | ENSG00000218336 |  | scz-gwas |  |  | 0 | 2 |
| *TMEM170B* | ENSG00000205269 |  | scz-gwas |  |  | 3 | 1 |
| *TMEM56* | ENSG00000152078 |  | scz-gwas |  |  | 3 | 2 |
| *VPS37A* | ENSG00000155975 |  | scz-gwas |  |  | 3 | 0 |
| *ZDHHC2* | ENSG00000104219 |  | scz-gwas |  |  | 1 | 3 |
| *ZNF804A* | ENSG00000170396 |  | scz-gwas |  |  | 0 | 0 |

**Table 2.** List of the genes influenced by genetic variants or IVs together with in/out-degrees

| **Gene** | **Symbol** | **IV** | **indegree** | **outdegree** | **degree*** |
| --- | --- | --- | --- | --- | --- |
| ENSG00000042781 | *USH2A* | 1 | 1 | 0 | 1 |
| ENSG00000066382 | *MPPED2* | 1 | 0 | 1 | 1 |
| ENSG00000074935 | *TUBE1* | 1 | 0 | 1 | 1 |
| ENSG00000083290 | *ULK2* | 1 | 0 | 0 | 0 |
| ENSG00000101346 | *POFUT1* | 2 | 0 | 2 | 2 |
| ENSG00000102290 | *PCDH11X* | 1 | 1 | 0 | 1 |
| ENSG00000102554 | *KLF5* | 1 | 0 | 0 | 0 |
| ENSG00000111224 | *PARP11* | 1 | 0 | 1 | 1 |
| ENSG00000112242 | *E2F3* | 1 | 0 | 1 | 1 |
| ENSG00000113119 | *TMCO6* | 1 | 0 | 1 | 1 |
| ENSG00000116120 | *FARSB* | 1 | 1 | 2 | 3 |
| ENSG00000119943 | *PYROXD2* | 1 | 0 | 0 | 0 |
| ENSG00000120324 | *PCDHB10* | 1 | 0 | 0 | 0 |
| ENSG00000126603 | *GLIS2* | 1 | 2 | 1 | 3 |
| ENSG00000126814 | *TRMT5* | 1 | 2 | 2 | 4 |
| ENSG00000132688 | *NES* | 1 | 0 | 1 | 1 |
| ENSG00000135679 | *MDM2* | 1 | 1 | 3 | 4 |
| ENSG00000137411 | *VARS2* | 1 | 0 | 0 | 0 |
| ENSG00000138621 | *PPCDC* | 1 | 0 | 0 | 0 |
| ENSG00000139289 | *PHLDA1* | 1 | 2 | 1 | 3 |
| ENSG00000140403 | *DNAJA4* | 1 | 1 | 1 | 2 |
| ENSG00000146757 | *ZNF92* | 1 | 1 | 2 | 3 |
| ENSG00000154813 | *DPH3* | 1 | 2 | 0 | 2 |
| ENSG00000157617 | *C2CD2* | 1 | 1 | 1 | 2 |
| ENSG00000159239 | *C2orf81* | 1 | 0 | 0 | 0 |
| ENSG00000161010 | *C5orf45* | 1 | 1 | 2 | 3 |
| ENSG00000165659 | *DACH1* | 1 | 2 | 0 | 2 |
| ENSG00000167280 | *ENGASE* | 1 | 1 | 1 | 2 |
| ENSG00000169231 | *THBS3* | 1 | 1 | 0 | 1 |
| ENSG00000169962 | *TAS1R3* | 1 | 0 | 0 | 0 |
| ENSG00000171724 | *VAT1L* | 1 | 1 | 0 | 1 |
| ENSG00000173273 | *TNKS* | 1 | 2 | 0 | 2 |
| ENSG00000175213 | *ZNF408* | 1 | 2 | 0 | 2 |
| ENSG00000181192 | *DHTKD1* | 1 | 3 | 0 | 3 |
| ENSG00000182054 | *IDH2* | 1 | 1 | 0 | 1 |
| ENSG00000185215 | *TNFAIP2* | 1 | 1 | 0 | 1 |
| ENSG00000187054 | *TMPRSS11A* | 1 | 1 | 0 | 1 |
| ENSG00000189050 | *RNFT1* | 1 | 1 | 0 | 1 |
| ENSG00000189164 | *ZNF527* | 1 | 3 | 0 | 3 |
| ENSG00000198039 | *ZNF273* | 1 | 3 | 0 | 3 |
| ENSG00000204348 | *DOM3Z* | 2 | 2 | 0 | 2 |
| ENSG00000205746 | *.* | 1 | 1 | 0 | 1 |
| ENSG00000218739 | *.* | 1 | 3 | 0 | 3 |
| ENSG00000220785 | *MTMR9LP* | 1 | 0 | 0 | 0 |
| ENSG00000229325 | *ACAP2-IT1* | 1 | 2 | 2 | 4 |
| ENSG00000230046 | *BIRC6-AS1* | 1 | 1 | 0 | 1 |
| ENSG00000232682 | *.* | 1 | 0 | 0 | 0 |
| ENSG00000232795 | *SCAND3P1* | 1 | 0 | 0 | 0 |
| ENSG00000233087 | *.* | 1 | 0 | 1 | 1 |
| ENSG00000233508 | *.* | 2 | 0 | 0 | 0 |
| ENSG00000234883 | *MIR155HG* | 1 | 2 | 0 | 2 |
| ENSG00000235531 | *.* | 2 | 2 | 1 | 3 |
| ENSG00000236064 | *.* | 1 | 0 | 0 | 0 |
| ENSG00000236558 | *.* | 1 | 0 | 1 | 1 |
| ENSG00000249550 | *.* | 1 | 1 | 0 | 1 |
| ENSG00000249568 | *.* | 1 | 1 | 1 | 2 |
| ENSG00000256306 | *.* | 1 | 2 | 0 | 2 |
| ENSG00000257647 | *.* | 2 | 0 | 0 | 0 |
| ENSG00000259030 | *FPGT-TNNI3K* | 1 | 2 | 0 | 2 |
| ENSG00000260118 | *.* | 2 | 0 | 0 | 0 |
| ENSG00000260267 | *.* | 1 | 2 | 0 | 2 |
| ENSG00000260331 | *.* | 1 | 0 | 0 | 0 |
| ENSG00000260400 | *.* | 1 | 1 | 0 | 1 |
| ENSG00000260743 | *.* | 1 | 2 | 0 | 2 |
| ENSG00000260807 | *.* | 1 | 2 | 0 | 2 |
| ENSG00000260942 | *.* | 1 | 0 | 0 | 0 |
| ENSG00000264198 | *.* | 4 | 1 | 0 | 1 |
| ENSG00000267796 | *LIN37* | 2 | 1 | 0 | 1 |
| ENSG00000267940 | *.* | 1 | 3 | 0 | 3 |
| ENSG00000269614 | *.* | 1 | 1 | 0 | 1 |
| ENSG00000269624 | *.* | 1 | 1 | 0 | 1 |

*degree: indegree+ outdegree

**Table 3.** Loss of mediator genes with biased expression in the human brain and their upstream and downstream genes in the network

| *Loss of mediators with biased expression in the human brain* | | | | | |
| --- | --- | --- | --- | --- | --- |
| Mediator Gene Symbol | **Entrez Gene Name** | **Chr** | **Expression** | **Downstream gene** | **Upstream Genes** |
| *GABRA2* | gamma-aminobutyric acid type A receptor alpha2 subunit | 4 | Biased expression in brain | *FUT9* | *ALDH5A1, PTPRG, GTF3C5* |
| *LRRTM2* | leucine rich repeat transmembrane neuronal 2 | 5 | Biased expression in brain | *MYO5B* | *CDKL5, MAGI3, DIEXF* |
| *PPM1E* | protein phosphatase, Mg2+/Mn2+ dependent 1E | 17 | Biased expression in brain | *MYT1L* | *AMPH, CLVS2* |

**Table 4.** Interactions of the proteins encoded by the loss of mediator genes and upstream and downstream genes in Table 3

**Table 5.** Genes with loss of mediator expressed in different human tissues together with their downstream and upstream genes

|  | *Loss of mediators* | | | | |
| --- | --- | --- | --- | --- | --- |
| Gene Symbol | **Entrez Gene Name** | **Chr** | **Expression** | **Downstream**  **gene** | **Upstream**  **genes** |
| SORT1 | sortilin 1 | 1 | Broad  (highest expression in brain) | *FBXO32* | *FNIP2, STARD13* |
| *GNAL* | G protein subunit alpha L | 18 | Ubiquitous  (highest expression in brain) | *CHRNA7* | *CHRM3, EPHA6* |
| *ZNF692* | zinc finger protein 692 | 1 | Ubiquitous  (highest expression in testis) | *HAUS5* | *AKT2, DNASE1L2* |
| RALGPS2 | Ral GEF with PH domain and SH3 binding motif 2 | 1 | Broad  (highest expression in testis) | *MMP16* | *RSBN1, EPHA7* |
| *ZNF672* | zinc finger protein 672 | 1 | Ubiquitous  (small differences among different tissues) | *GET4* | *LZTS2, BRCC3* |
| *SNRNP48* | small nuclear ribonucleoprotein U11/U12 subunit 48 | 6 | Ubiquitous  (small differences among different tissues) | *MLLT3* | *ARSK, CHORDC1* |

**Table 6.** Interactions of the proteins encoded by the loss of mediator genes and their upstream and downstream genes in Table 5
