# Supplementary material for: Differential gene regulatory pattern in the human brain from schizophrenia using transcriptomic-causal network
Source: BMC Bioinformatics. 2020 Oct 21;21:469. doi: 10.1186/s12859-020-03753-6 (PMC7579819; doi:10.1186/s12859-020-03753-6)
Supplement: Supplementary file 2 — Additional file 2: Figure 1. A: Circos plot represents the interaction of the genes from different chromosomes; B: Pie-chart of the replication analysis after FDR adjustment. [file 12859_2020_3753_MOESM2_ESM.docx]

**Differential gene regulatory pattern in the human brain from schizophrenia using transcriptomic-causal network**

Akram Yazdani*, Raul Mendez-Giraldez, Azam Yazdani, Michael R Kosorok, Panos Roussos*

**Additional file 2: Figures**

**Figure 1. A**: Circos plot represents the interaction of the genes from different chromosomes; **B**: Pie-chart of the replication analysis after FDR adjustment
