# Supplementary material for: Differential gene regulatory pattern in the human brain from schizophrenia using transcriptomic-causal network
Source: BMC Bioinformatics. 2020 Oct 21;21:469. doi: 10.1186/s12859-020-03753-6 (PMC7579819; doi:10.1186/s12859-020-03753-6)
Supplement: Supplementary file 3 — Additional file 3: Modules. [file 12859_2020_3753_MOESM3_ESM.docx]

**Differential gene regulatory pattern in the human brain from schizophrenia using transcriptomic-causal network**

Akram Yazdani*, Raul Mendez-Giraldez, Azam Yazdani, Michael R Kosorok, Panos Roussos*

**Additional file 3: Modules**

In the following, we explored few modules in our network depicted as a sub-network in the corresponding figures for simplicity. For each module, the strength of the regulatory interactions in the network is represented by a heatmap of their corresponding p-values above a certain cutoff value, that is defined as the significance level of the likelihood for an edge to connect two genes (regulatory interaction) given the other genes in the network.

***TENM3*-Module:** *TENM3* had previously associated to SCZ with impact on the transcription of 9 other genes downstream in our network (Supplementary Table 1), 6 of which were known to be related to SCZ (Figure 1A, 1C). We explored the impact of *TENM3*, considered as the hub for this module, on non-hub genes within the module by predicting their transcription levels from *TENM3* using cross-validation. Figure 1A represents the module or sub-network for controls. The prediction analysis resulted in a very good performance in both SCZ and control groups (Figure 1B). Although *TENM3* predicts slightly better the transcription level for the genes in the control (blue) as compared to the SCZ group (red), the result strongly supports the interaction of hub with non-hub genes in both cases and controls. Therefore, *TENM3* plays a key role in controlling the transcription level of *BTRC*, *RNF150*, *SEZ6L*, *GRIN3A*, *VAT1L*, *GRIA1*, and *XKR4* SCZ-associated genes.

**Table 1.** *TENM3*-Module genes information

| *TENM3*-Module | | | | |
| --- | --- | --- | --- | --- |
| Gene Symbol | **Entrez Gene Name** | **Location** | **Type(s)** | **Drug(s)** |
| *BTRC* | beta-transducin repeat containing E3 ubiquitin protein ligase | Cytoplasm | enzyme | -- |
| *GRIA1* | glutamate ionotropic receptor AMPA type subunit 1 | Plasma Membrane | ion channel | desflurane,enflurane,farampator,isoflurane,  LY451395,methoxyflurane,perampanel,  sevoflurane,talampanel,tezampanel |
| *GRIN3A* | glutamate ionotropic receptor NMDA type subunit 3A | Plasma Membrane | ion channel | 1-aminocyclopropane-1-carboxylic acid, amantadine,amitriptyline/ketamine, aspirin/caffeine/orphenadrine, besonprodil, bicifadine, brompheniramine/dextromethorphan/pseudoephedrine,  bupropion/dextromethorphan, caffeine/ethanol, carbinoxamine/dextromethorphan/pseudoephedrine,  chlorpheniramine/dextromethorphan/phenylephrine, cycloserine, delucemine, dextromethorphan, dextromethorphan/morphine,dextromethorphan/promethazine,  dextromethorphan/quinidine,donepezil/memantine, ethanol, ethopropazine, felbamate, ketamine, memantine, methadone, N-(2-indanyl) glycinamide, nebostinel, neramexane, orphenadrine, phencyclidine, procaine, tenocyclidine,UK-240455 |
| *RNF150* | ring finger protein 150 | Other | peptidase | -- |
| *SEZ6L* | seizure related 6 homolog like | Plasma Membrane | other | -- |
| *SORCS3* | sortilin related VPS10 domain containing receptor 3 | Nucleus | transporter | -- |
| *TCP11L1* | t-complex 11 like 1 | Cytoplasm | other | -- |
| *TENM3* | teneurin transmembrane protein 3 | Plasma Membrane | other | -- |
| *VAT1L* | vesicle amine transport 1 like | Other | enzyme | -- |
| *XKR4* | XK related 4 | Other | other | -- |

**
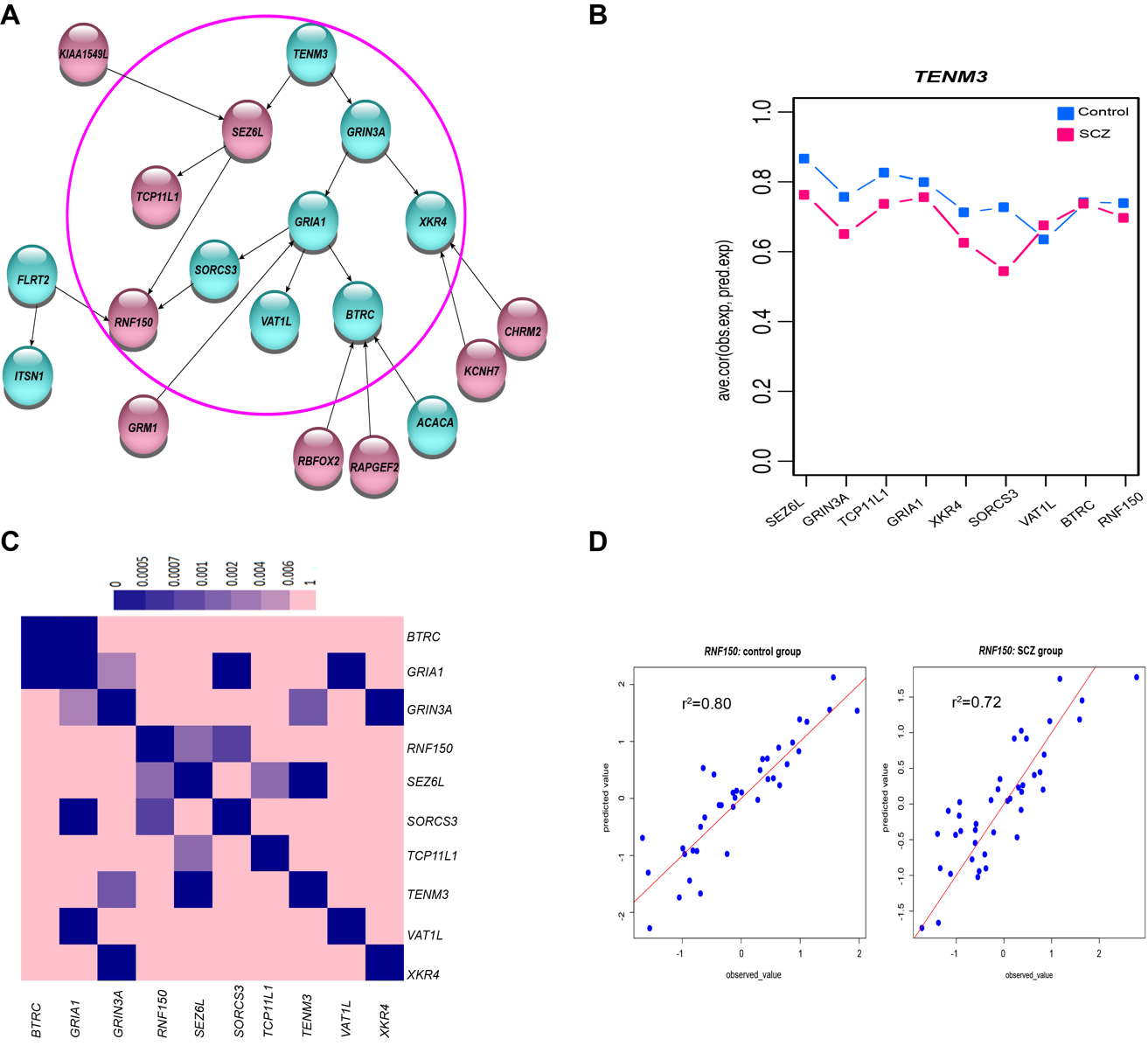
**

**Figure 1**. **A**: *TENM3*-module with SCZ-associated genes in cyan. **B**: Prediction of non-hub gene transcription levels based on transcription of the hub. This plot shows the average Pearson correlation coefficient between observed and predicted values for controls (blue) and SCZ individuals (red). **C**: Heatmap of the strength of the relationship between genes in the modules based on a p-value cut-off. **D**: Prediction of *RNF150* transcription level in both SCZ and control groups using *SEZ6L, FLRT2, SORCS3* transcription level as predictor.

The longest path in this module is *TENM3*🡪 *GRIN3A*🡪 *GRIA1*🡪 *SORCS3*🡪 *RNF150*. All the genes in this pathway are associated with SCZ except *RNF150*. Therefore, we hypothesized that *RNF150* is a SCZ-associated gene as well. This hypothesis is supported by the fact that another SCZ-associated gene from out of the module, FLRT2, directly affects *RNF150* transcription. We predicted *RNF150* transcription level based on the transcription of all three genes (*SORCS3, FLRT2, SEZ6L*) with direct impact on *RNF150* using a penalization-regression model and cross-validation (see methods for details). These three genes explained more than 70% of the *RNF150* transcription variance in both groups (Figure 1D), which agrees with our hypothesis since one of the two upstream genes of *SEZ6L* is SCZ-associated gene as well. Therefore, we propose *RNF150* as a SCZ candidate gene.

We used the Human Protein Atlas (HPA) to investigate the tissue-specific expression of genes in this module, both at mRNA and protein levels (Figure 2). All the genes are expressed to some extent in the human brain, although for some of them (*SEZ6L*, GRIN3A, *XKR4,* and *SORCS3*) there were no data available regarding the protein levels. The hub of the module, *TENM3,* showed high transcription and low protein level in the human brain. It affected directly the transcription of two genes that are also highly transcribed in the brain (*SEZ5L* and *GRIN3A*). A search into the STRING database for physical interactions between the proteins coded by those genes with evidence of expression (GRIA1, BTRC, TCP11L1, RNF150) revealed the following interaction pairs: GRIA1– BTRC (mediated by DLG1), BTRC – ACACA (mediated by GSK3B) and BTRC – RAPGEF2 (mediated by CTNNB1). All these interactions are based on experimental evidence (Table 2).

**Figure 2.** Tissue-specific expression from Human Protein Atlas (HPA)

**Table 2:** Interaction of proteins encoded by the genes in *TENM3*-module

***NRXN3*-Module:** The NRXN3-module (Figure 3A, 3C) includes 4 genes (*ARFGEF3*, *HECW2*, *DLGAP1*, *CNTNAP2*) whose transcription are directly influenced by *NRXN3* hub (Table 3). Even though the effect of *NRXN3* itself does not propagate further within the network after one step, we believe it is an important gene since it encodes a member of a family of proteins that function in the nervous system as receptors and cell adhesion molecules. *NRXN3* is preferentially transcribed in the brain according to HPA and has also been found associated with SCZ in a Genome-Wide Association Study (GWAS) (1). This module unveils that *MAS1* affects *NRXN3* and *FREM3* transcription, but the impact on *NRXN3* is much higher (Figure 3C)*. NRXN3* exerts the effect of *MAS1* into the system although *FRM3* blocks the effect of *MAS1*. Interestingly, Gene Ontology analysis shows that all genes in the module except *FRM3* are related to “cellular development, organismal injury, and abnormalities”.

**Table 3.** *NRXN3*-Module genes information

| *NRXN*3-Module | | | | | |
| --- | --- | --- | --- | --- | --- |
| Gene Symbol | **Entrez Gene Name** | **Location** | **Type(s)** | **Drug(s)** | |
| *ARFGEF1* | ADP ribosylation factor guanine  nucleotide exchange factor 1 | Cytoplasm | other | -- | |
| *CNTNAP2* | contactin associated protein like 2 | Plasma Membrane | other | -- | |
| *FREM3* | FRAS1 related extracellular matrix 3 | Extracellular Space | other | -- | |
| *HECW2* | HECT, C2 and WW domain containing  E3 ubiquitin protein ligase 2 | Extracellular Space | enzyme | -- | |
| *MAS1* | MAS1 proto-oncogene,  G protein-coupled receptor | Plasma Membrane | G-protein coupled receptor | | -- |
| *NRXN3* | neurexin 3 | Plasma Membrane | transporter | -- | |

We predicted gene transcription levels in both SCZ and control groups based on *NRXN3* transcription. Figure 3B shows the average of the Pearson correlation coefficients between predicted and observed transcription values for SCZ (red) and control (blue) groups. The high agreement between predictions of downstream transcription levels in both, SCZ cases and controls, is evidence of the essential role of *NRXN3* in brain functioning.


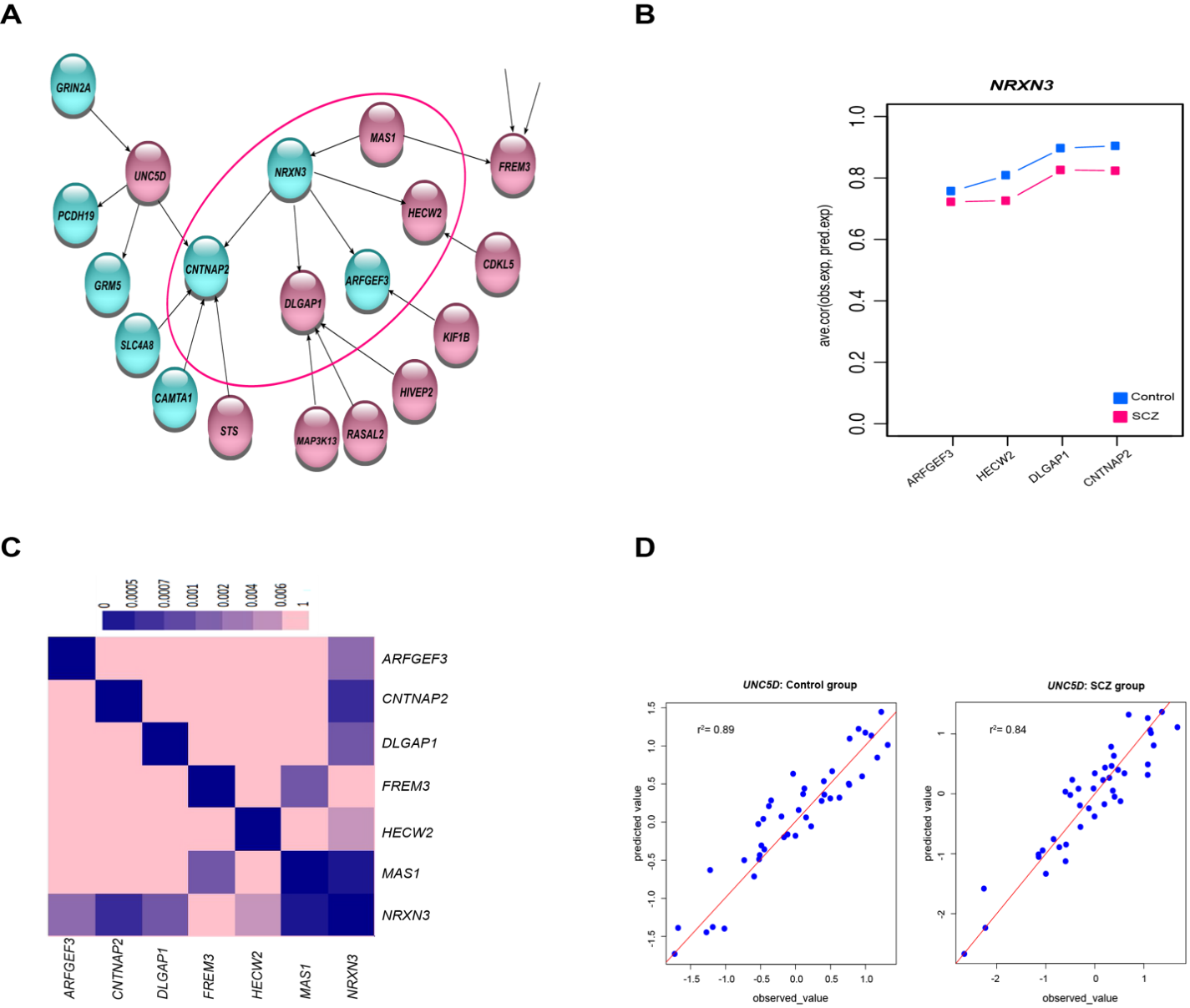


**Figure 3**. **A:** *NRXN3*-module with previously SCZ-associated genes in cyan. **B**: Prediction of non-hub gene transcription levels based on transcription of the hub. The plot shows the average Pearson correlation coefficient between expected and predicted transcription. **C**: Heatmap of the strength of the relationship between genes in the modules based on the significance of their association in the network. **D**: Prediction *UNC5D* transcription level in both SCZ and control groups using *GRIN2A, PCDH19, GRM5, CNTNAP2* transcription levels.

The highest impact of *NRXN3* is on *CNTNAP2* gene (Figure 3A, 3C) which is influenced by four genes (*UNC5D*, *SLC4A8*, *CAMTA1,* and *STS*) from outside the module. Among these, *UNC5D* is the most relevant to our study, since its transcription directly influenced three SCZ-associated genes (*PCDH19, GRM5, CNTNAP2*) and it is also influenced by another SCZ-associated gene (*GRIN2A*). Therefore, we hypothesized that *UNC5D* is a gene related to SCZ, given that *PCDH19, GRM5,* and *CNTNAP2* are associated with SCZ. We tested this hypothesis by predicting the transcription level of *UNC5D* as a linear outcome of the genes with a direct connection (*GRIN2A, PCDH19, GRM5,* and *CNTNAP2*). As can be seen in Figure 3D, the high predictions in both cases and controls support our hypothesis. We also searched for protein-protein interactions in the STRING database and found strong indirect interaction between the proteins UNC5D and GRIN2A detailed in Table 4.

According to the HPA, *NRXN3* is moderately expressed in the brain at both mRNA and protein levels (Figure 4). Among the four genes that are directly affected by the hub, *CNTNAP2* and *HECW2* show also high protein and mRNA levels in the brain. Although there is no data available for *DLGAP1* and *ARFGEF3* at the protein level in HPA, *DLGAP1* is one of the genes with the highest mRNA level in the module with primary expression in the human brain tissue. Searching for protein-protein interactions in STRING database, we found the following interactions: NRXN3 – DLGAP1; NRXN3 – CNTNAP2 (mediated by CASK); GRIN2A – PCDH19; GRIN2A – CNTNAP2; GRIN2A – GRM5. More details can be found in Table 4.

**Figure 4.** Tissue-specific expression from Human Protein Atlas (HPA)

**Table 4:** Interaction of proteins encoded by the genes in *NRXN3*-module

***MYH10*-Module**: *MYH10* is a SCZ-associated gene that influences the transcription of 7 other genes in the network (Figure 5A and Table 5). Its transcription is influenced seemingly by two upstream genes (*ADAM23* and *CHD6)*. In this module, all the genes except those influenced by *MYH10* through *LMO7* pathway are associated with SCZ. *MYH10* and *LMO7* in turn, are in the longest and strongest path in this module (Figure 5A, 5C). The impact of the hub on the downstream genes is investigated further using the prediction model. The transcription of all target genes in the module can be inferred from the transcription activity of the *MHYH10* in SCZ-cases and controls, except for the furthest-related (*HMG20A*) gene to the hub (Figure 5B). Consequently, we hypothesized that *LMO7* and *RTF1* are also associated with SCZ. We assessed the effect of *LMO7* on *RTF1* to see if this influence is due to *MYH10* that is a SCZ-associated gene or on the contrary, it is due to *ROCK2*. Using conditional analysis, we calculated the exclusive effect regarding *MYH10*, *ROCK2* and both (Figure 5D). Hence, *LOM7* and *RTF1* both are associated with SCZ. Furthermore, we conclude that *MYH10* has a key role in regulating the transcription of *ANK2, LMO7, LRRC4C, DLG2, RTF1* and *PTPRK* genes.


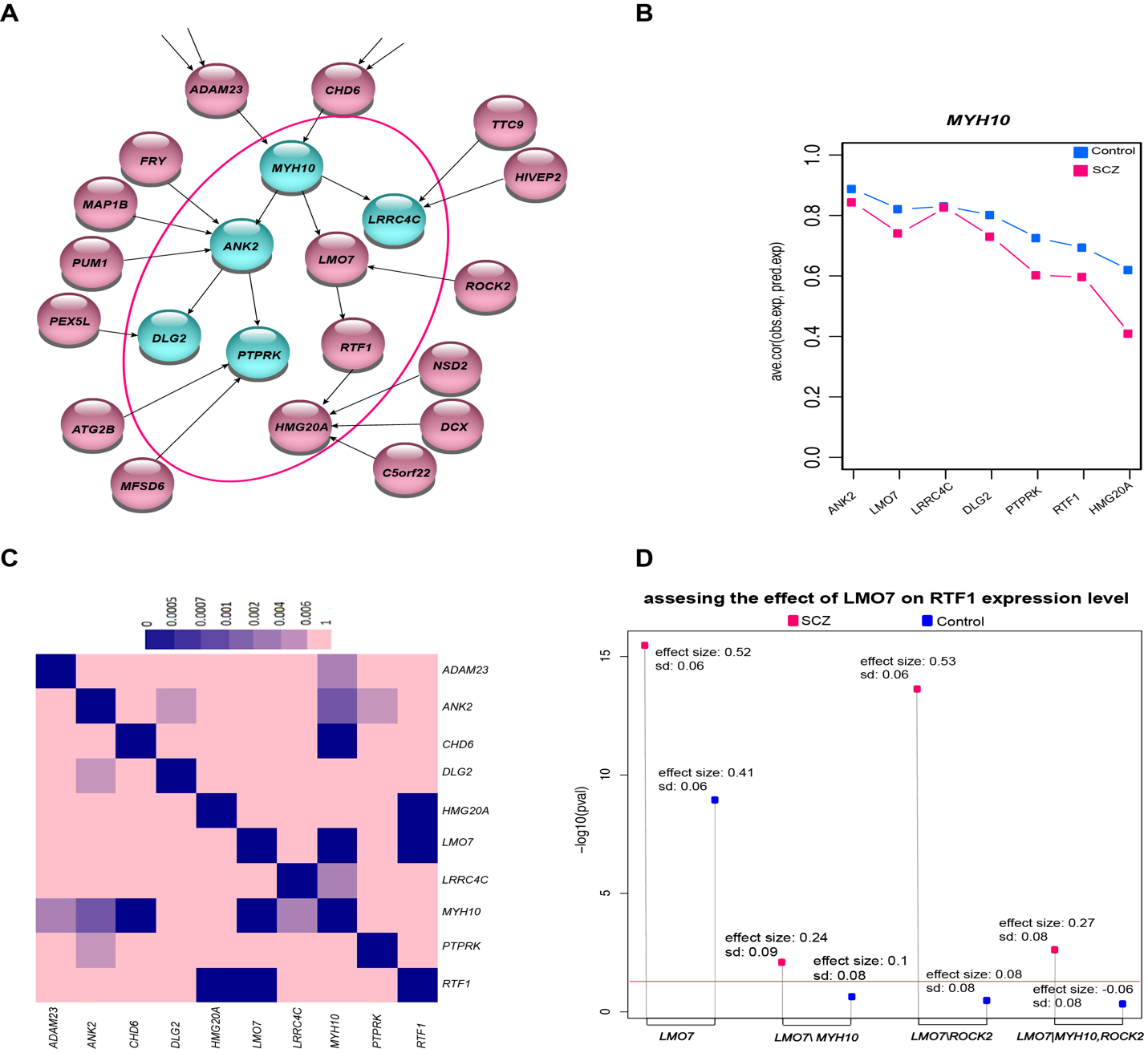


**Figure 5**. **A**: *MYH10*-module with SCZ-associated genes in cyan. **B**: Prediction of non-hub gene transcription levels based on transcription of the hub. The plot shows the average Pearson correlation coefficient between expected and predicted transcription. **C**: Heatmap of the strength of the relationship between genes in the modules based on the significance of their association in the network. **D**: Summary-result of assessing the effect of *LMO7* on *RTF1* using conditional analysis in 4 different scenarios.

All the genes included in the *MYH10*- module are expressed significantly in the brain, at both mRNA and protein levels (Figure 6). STRING database showed the protein interactions of MYH10 - ANK2 (mediated by MYH9 and SPTAN1), ANK2 - DLG2 (mediated by NRCAM or NFASC), LRRC4C - DLG2 and MYH10 - RTF1 (mediated by MYH9 and CDC5L). MYH10 protein and its upstream donor in our network ADAM23, which shows medium protein level in the brain according to HPA, interact through RHOA and DLG4 mediators (Table 6). In addition, Gene Ontology analysis categorized all genes in *MYH10*-module with similar functions, “cell-to-cell signaling and interaction, nervous system development and function, cell morphology”.

**Table 3.** *MYH10*-Module genes information

| *MYH10*-Module | | | | |
| --- | --- | --- | --- | --- |
| Gene Symbol | **Entrez Gene Name** | **Location** | **Type(s)** | **Drug(s)** |
| *ANK2* | ankyrin 2 | Plasma Membrane | other | -- |
| *DLG2* | discs large MAGUK scaffold protein 2 | Plasma Membrane | kinase | -- |
| *HMG20A* | high mobility group 20A | Nucleus | Transcription  regulator | -- |
| *LMO7* | LIM domain 7 | Cytoplasm | enzyme | -- |
| *LRRC4C* | leucine rich repeat containing 4C | Plasma Membrane | other | -- |
| *MYH10* | myosin heavy chain 10 | Cytoplasm | enzyme | -- |
| *PTPRK* | protein tyrosine phosphatase, receptor type K | Plasma Membrane | phosphatase | -- |
| *RTF1* | RTF1 homolog, Paf1/RNA polymerase II  complex component | Nucleus | other | -- |

**Figure 6.** Tissue-specific expression from Human Protein Atlas (HPA)

**Table 6:** Interaction of proteins encoded by the genes in *MYH10*-module

***PEX5L*-Module**: *PEX5L* with high protein and expression level in brain (Figure 7) has a strong significant impact on two genes associated with SCZ (*STARD13* and *DLG2*) (Figure 8A, 8C and Table 7) making *PEX5L* a SCZ candidate target. The effect of *PEX5L* is very strong upon the genes of the first interaction shell and it decreases along the path through successive indirect interactions (Figure 8C). *PEX5L* lost its indirect effect on *FBXO32, ZBTB43,* and *TGOLN2* genes in SCZ-cases as compared to controls since it shows poor prediction performance for these genes for SCZ-cases (Figure 8B).

**Figure 7.** Tissue-specific expression from Human Protein Atlas (HPA)

*DLG2* is a receptor at the edge of the *PEX5L*- and *MYH10*-modules. Although the strength of connection in the former module is higher than in the latter, both hubs are good predictors for *DLG2* transcription level. Therefore, *DGL2* regulation takes place through either the *PEX5L*- or the *MYH10*-module (or both simultaneously) in transcription level. However, it may not hold in protein level since protein-protein interaction data from STRING shows DLG2 has strong interaction with MYH10 protein through ANK2 (Table 8) and no evidence of protein–protein interactions with PEXL5.

Figure 8A also shows that the direction of *PALM2* interaction over *PEX5L* could not be identified through this analysis. While *PALM2* is out of the module, at the edge of the absolute network and has no genetic variants associated with it, we believe that *PALM2* transcription may be regulated by other genes that are not included in this study. All genes in this module share the same function related to “Cellular development, cellular Growth and proliferation, nervous system development and function” according to the Gene Ontology.


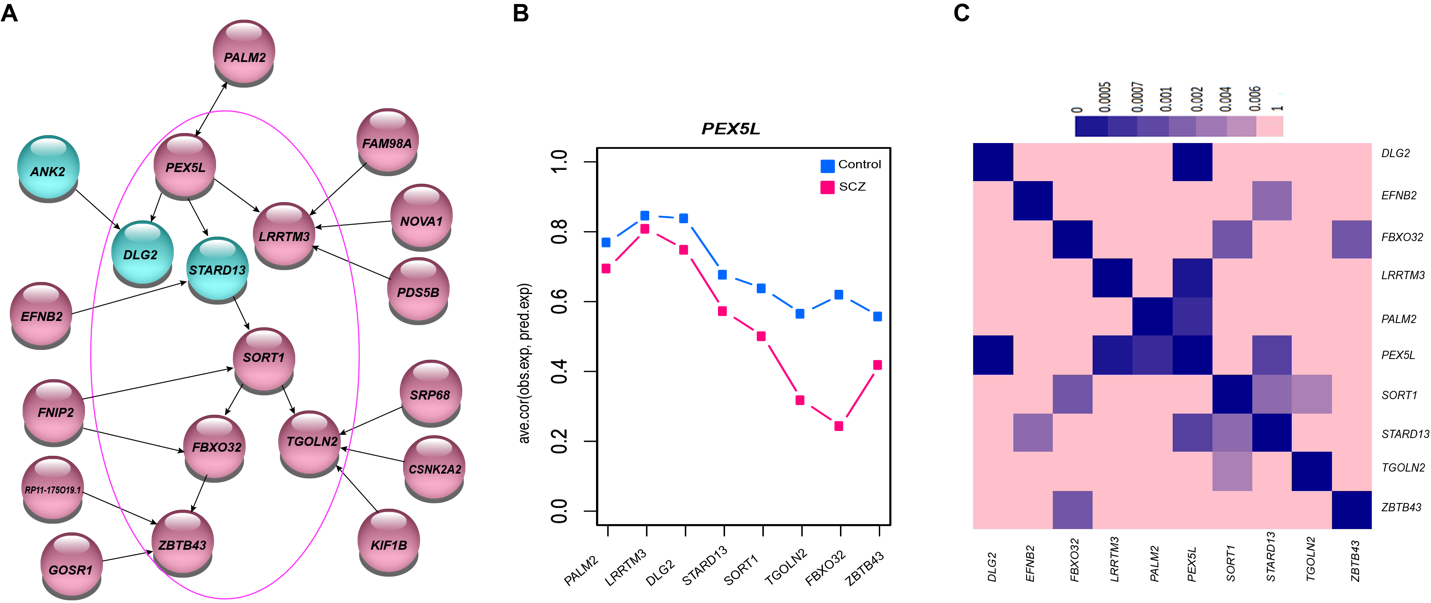


**Figure 8. A**: PEX5L-module with a SCZ-associated in cyan. **B**: Prediction of non-hub gene transcription levels based on transcription of the hub. The plot shows the average Pearson correlation coefficient between expected and predicted transcription. **C**: Heatmap of the strength of the relationship between genes in the modules based on the significance of their association in the network.

Three genes, *GABRA2*, *LRRTM2* and *PPM1E* that are differentially regulated in individuals diagnosed with SCZ are also preferentially expressed in the human brain. All these genes are related to the synapse, which have been hypothesized to underlie altered neuronal function in complex neuropsychiatric disorders (2). In particular, *GABRA2* is a component of the hetero-pentameric receptor for GABA, the major inhibitory neurotransmitter in the brain. Consequently, it plays an important role in the formation of functional inhibitory GABAergic synapses in addition to mediating synaptic inhibition as a GABA-gated ion channel (based on homology). *LRRTM2* is involved in the development and maintenance of the excitatory synapse in vertebrates, regulates surface expression of AMPA receptors, and instructs the development of functional glutamate release sites (3). *PPM1E* encodes protein phosphatase that inactivates multifunctional CaM kinases, whose activation can affect many downstream pathways controlling a variety of cellular functions (4).

**Table 7.** *PEX5L*-Module genes information

| *PEX5L*-Module | | | | |
| --- | --- | --- | --- | --- |
| Gene Symbol | **Entrez Gene Name** | **Location** | **Type(s)** | **Drug(s)** |
| *DLG2* | discs large MAGUK scaffold protein 2 | Plasma Membrane | kinase | -- |
| *FBXO32* | F-box protein 32 | Cytoplasm | enzyme | -- |
| *Fbxo32* | -- | Other | other | -- |
| *LRRTM3* | leucine rich repeat transmembrane  neuronal 3 | Plasma Membrane | other | -- |
| *PEX5L* | peroxisomal biogenesis factor 5 like | Cytoplasm | ion channel | -- |
| *SORT1* | sortilin 1 | Plasma Membrane | G-protein coupled receptor | -- |
| *STARD13* | StAR related lipid transfer domain  containing 13 | Cytoplasm | other | -- |
| *TGOLN2* | trans-golgi network protein 2 | Cytoplasm | other | -- |
| *ZBTB43* | zinc finger and BTB domain containing 43 | Nucleus | transcription regulator | -- |

Our analysis shows differential regulation of *GNAL* between SCZ and control groups*.* In our network, *GNAL* that encodes a stimulatory G protein alpha subunit is a mediator between two SCZ drug targets (*CHRM3* and CHRNA7). The protein coded by the upstream gene *CHRM3* is targeted by antipsychotic drugs against schizophrenia and bipolar disorder. *CHRNA7* as a downstream gene of *GNAL* is also considered a promising drug target for the treatment of cognitive dysfunction in schizophrenia and improves memory and executive functions in patients and healthy individuals. However, clinical trials with pro-cognitive drugs are challenged by large inter-individual response variations. This differential regulatory pattern suggests that *GNAL* can be an alternative SCZ drug target since it encodes a G protein alpha subunit that is widely expressed in the central nervous system.

**Table 6:** Interaction of proteins encoded by the genes in *PEX5L*-module

The following four genes *PEX5L, TENM3, NRXN3, MYH10* are essential for brain function since they have high impacts on several genes in both SCZ and control groups. *PEX5L* has a biased expression in the brain. The corresponding PEX5L protein is believed to work as an accessory subunit of hyperpolarization-activated cyclic nucleotide-gated channels, regulating their cell-surface expression and cAMP dependence (5). *PEX5L* exerts a significant effect directly on the transcription of three genes: *DGL2, STARD13,* and *LRRTM3*. While the first two had been previously linked to SCZ, *LRRTM3* shows biased expression in the brain. Homologous proteins to LRRTM3 are involved in the development and maintenance of the vertebrate nervous system (5). Furthermore, among all downstream genes of *PEX5L*, *FBXO32* shows the highest difference in regulation between cases and controls. This differential pattern is supported by the fact that FBXO32 protein promotes neuronal protein homeostasis through coordinating autophagy/lysosome-mediated protein turnover with the ubiquitin–proteasome system (6).

*TENM3* encodes a protein that translocates in the nucleus, regulating the transcriptional activity of genes related to neurite growth and cell adhesion (7). This is clearly in agreement with our results since *TENM3* strongly affects multiple genes in our network. In addition, it has been shown that its homologous protein establishes the neuron connectivity in the mouse brain (8). NRXN3 protein is involved in synaptic plasticity (9). Moreover, among the four genes directly influenced by *NRXN3,* three of them *(DLGAP1, DLGAP1, CNTNAP2*) are related to synaptic plasticity as well (10, 11)(11). Thus, it makes sense to consider all five genes associated with synaptic dysfunction, which is a causal factor for neuropsychiatric disorders including SCZ. *MYH10* gene is important for the normal development and function of dendritic spines (12–15).

**References**

1. Hu,X., Zhang,J., Jin,C., Mi,W., Wang,F., Ma,W., Ma,C., Yang,Y., Li,W., Zhang,H., *et al.* (2013) Association study of NRXN3 polymorphisms with schizophrenia and risperidone-induced bodyweight gain in Chinese Han population. *Prog. Neuro-Psychopharmacology Biol. Psychiatry*, **43**, 197–202.

2. Wang,X., Christian,K.M., Song,H. and Ming,G. li (2018) Synaptic dysfunction in complex psychiatric disorders: From genetics to mechanisms. *Genome Med.*, **10**.

3. Medicine,N.L. of National Library of Medicine (US). *Natl. Libr. Med. (US). Genet. Home Ref.*

4. Swulius,M.T. and Waxham,M.N. (2008) Ca2+/calmodulin-dependent protein kinases. *Cell. Mol. Life Sci.*, **65**, 2637–2657.

5. The UniProt Consortium,U. (2017) UniProt: The universal protein knowledgebase. *Nucleic Acids Res.*, **45**, D158–D169.

6. Murdoch,J.D., Rostosky,C.M., Gowrisankaran,S., Arora,A.S., Soukup,S.F., Vidal,R., Capece,V., Freytag,S., Fischer,A., Verstreken,P., *et al.* (2016) Endophilin-A Deficiency Induces the Foxo3a-Fbxo32 Network in the Brain and Causes Dysregulation of Autophagy and the Ubiquitin-Proteasome System. *Cell Rep.*, **17**, 1071–1086.

7. Wikpedia contributors (2019) Teneurin. *n Wikipedia, Free Encycl.*

8. Berns,D.S., DeNardo,L.A., Pederick,D.T. and Luo,L. (2018) Teneurin-3 controls topographic circuit assembly in the hippocampus. *Nature*, **554**, 328–333.

9. Kelai,S., Maussion,G., Noble,F., Boni,C., Ramoz,N., Moalic,J.-M., Peuchmaur,M., Gorwood,P. and Simonneau,M. (2008) Nrxn3 upregulation in the globus pallidus of mice developing cocaine addiction. *Neuroreport*, **19**, 751–5.

10. Toro,R., Konyukh,M., Delorme,R., Leblond,C., Chaste,P., Fauchereau,F., Coleman,M., Leboyer,M., Gillberg,C. and Bourgeron,T. (2010) Key role for gene dosage and synaptic homeostasis in autism spectrum disorders. *Trends Genet.*, **26**, 363–372.

11. Ullman,N.L., Smith-Hicks,C.L., Desai,S. and Stafstrom,C.E. (2018) De Novo HECW2 Mutation Associated With Epilepsy, Developmental Decline, and Intellectual Disability: Case Report and Review of Literature. *Pediatr. Neurol.*, **85**, 76–78.

12. Zhang,H., Webb,D.J., Asmussen,H., Niu,S. and Horwitz,A.F. (2005) A GIT1/PIX/Rac/PAK signaling module regulates spine morphogenesis and synapse formation through MLC. *J. Neurosci.*, **25**, 3379–3388.

13. Ryu,J., Liu,L., Wong,T.P., Wu,D.C., Burette,A., Weinberg,R., Wang,Y.T. and Sheng,M. (2006) A critical role for myosin IIB in dendritic spine morphology and synaptic function. *Neuron*, **49**, 175–182.

14. Rex,C.S., Gavin,C.F., Rubio,M.D., Kramar,E.A., Chen,L.Y., Jia,Y., Huganir,R.L., Muzyczka,N., Gall,C.M., Miller,C.A., *et al.* (2010) Myosin IIb Regulates actin dynamics during synaptic plasticity and memory formation. *Neuron*, **67**, 603–617.

15. Hodges,J.L., Newell-Litwa,K., Asmussen,H., Vicente-Manzanares,M. and Horwitz,A.R. (2011) Myosin IIB activity and phosphorylation status determines dendritic spine and post-synaptic density morphology. *PLoS One*, **6**.
